# Reordered hierarchical complexity in ecosystems with delayed interactions

**DOI:** 10.1093/pnasnexus/pgaf214

**Published:** 2025-07-14

**Authors:** Bo-Wei Qin, Wenbo Sheng, Xuzhe Qian, Jürgen Kurths, Alan Hastings, Ying−Cheng Lai, Wei Lin

**Affiliations:** Research Institute of Intelligent Complex Systems, Fudan University, Shanghai 200433, China; Shanghai Artificial Intelligence Laboratory, Shanghai 200232, China; School of Mathematical Sciences, SCMS, and SCAM, Fudan University, Shanghai 200433, China; Research Institute of Intelligent Complex Systems, Fudan University, Shanghai 200433, China; School of Mathematical Sciences, SCMS, and SCAM, Fudan University, Shanghai 200433, China; Research Institute of Intelligent Complex Systems, Fudan University, Shanghai 200433, China; Potsdam Institute for Climate Impact Research, Potsdam 14412, Germany; Department of Environmental Science and Policy, University of California, One Shields Avenue, Davis, CA 95616, USA; Santa Fe Institute, 1399 Hyde Park Road, Santa Fe, NM 87501, USA; School of Electrical, Computer and Energy Engineering, and Department of Physics, Arizona State University, Tempe, AZ 85287-5706, USA; Research Institute of Intelligent Complex Systems, Fudan University, Shanghai 200433, China; Shanghai Artificial Intelligence Laboratory, Shanghai 200232, China; School of Mathematical Sciences, SCMS, and SCAM, Fudan University, Shanghai 200433, China

**Keywords:** ecosystem, delayed interactions, stability order, random matrix, network complexity

## Abstract

It was once believed that large ecosystems with random interactions are unstable, limiting their complexity. Thus, large community size or numerous interactions are rare in nature. Later, a strict hierarchical complexity was revealed: competitive and mutualistic communities have the least complexity, followed by random ones, and then predator–prey communities. Recently, a hierarchy of recovery times for ecosystems with identical complexity was found, influenced by discrete time delays. A key question is whether this hierarchical complexity holds under noninstantaneous interactions. We surprisingly show that it does not. Specifically, the complexity of predator–prey communities is significantly affected by time delays, reordering the hierarchy at a critical threshold. These changes exhibit nonmonotonic behavior with continuous time delays, another realistic interaction type. We validated our findings in various realistic ecosystems. Our results indicate that incorporating factors like time delays and their appropriate forms can lead to correct and even deeper understanding about complexity of large ecosystems and other biophysical systems.

Significance Statement Complexity and stability of ecosystems are of paramount importance to sustainability of the human society. Sir Robert May argued that sufficiently large ecosystems with random interactions are unstable. It was revealed later that various ecosystems can possess a strict hierarchical complexity. An open question is whether ecosystems can maintain the hierarchy of complexity under noninstantaneous interactions. This study develops a rigorous analysis, revealing a reordered hierarchy of complexity in a large variety of realistic ecosystems with time delayed interactions with the implication that it is unlikely to observe large, complex predator–prey type of ecosystems in nature. Our work provides fresh insights into the fundamental interplay between stability and complexity in ecosystems that are significantly more realistic than those studied previously.

## Introduction

Large and complex ecosystems are generally not susceptible to experiments, rendering analytic investigations through dynamical models fundamentally important for understanding and predicting their behaviors. The stability bounds of equilibrium abundances for various ecological communities are of great significance and interests. Here, we refer to the stability as the local asymptotic stability of the equilibrium abundances that characterize the community’s ability to recover from external perturbations. Therefore, it is critical not only to the fundamental issues of persistence and extinction but also to practical problems encompassing the admissible complexity, energy cost, recovery time, and their trade-off associated with certain control strategies. A seminal result is May’s stability bound: for large ecosystems with random communities, there is a maximal admissible complexity to maintain stable abundances (1, 2).

In a closely related work (3), different types of communities, such as predator–prey, mutualism or competition, were considered with the analytic finding that different communities exhibit different stability bounds. A key result was the emergence of an ordering of the admissible complexities of different communities characterizing the capacity, the number and uncertainties of the interactions. Particularly, it was found (3) that predator–prey communities allow the largest complexity, while a mixture of mutualistic and competitive interactions accommodate the least one, and the fully random ecosystems is somewhere in the middle. May’s complexity–stability trade-off was demonstrated lately without knowing the underlying interactions (4, 5), and the system’s behavior beyond the transition to instability was addressed (6). Quite recently, an inverse approach to modeling food webs was articulated (7), where it was assumed that stable food webs exist and the goal was to identify the characteristics of such systems. The work afforded comprehensive insights into how biodiversity promotes ecological stability and how nature may respond to growing anthropogenic disturbances.

Besides complexity, time delay is also an ubiquitous and critical factor impacting stability. Interactions among species are typically not instantaneous, such as a latency period of maturation in population dynamics (8) and delayed predation in ecosystems (9). The effect of delay is generally modeled as discrete or continuous time (10–13). An earlier work showed that continuous time delay delineating age-dependent predation alters the stability of a 2D ecosystem (14). Later, the significance of the variance of continuous delays for stabilizing the system was revealed (15). For large and complex ecosystems, a comprehensive result on how time delay affects complexity–stability trade-off is still lacking, though there were works derived some related results.

Previous works showed that May’s stability bound holds even when there are different types of time delay (16, 17), but there were studies providing the contrary result that, in oscillatory ecosystems, time delays change the stability criteria (18–20). Recently, it was demonstrated that the stability bound of a random community changes dramatically when considering delayed self-interactions (21). More broadly, time delays can regulate collective dynamics in dynamical networks, such as synchronization (22, 23) and also yield oscillatory and chaotic phenomena (24–26). Regarding the hierarchical order, it was uncovered recently that a discrete-time delay modulates significantly the order of the heuristically estimated recovery time of different types of ecosystems (27). Specifically, when time delay is small, the communities with predator–prey interactions exhibit the least recovery time followed by the random community, and then those communities with purely competitive and mutualistic pairs. The order of recovery times changes as time delay increases (27). The recovery time is one of the metrics delineating the ecological stability. However, it does not inform us how admissible complexity of a particular ecosystem changes after introducing time delay, which may be very different from the behavior of the recovery rate. More importantly, how prolonged effect such as “ecological memory” influence the stability of large ecosystems is still an open question.

Establishing rigorous stability bounds for large ecosystems with time delays presents significant analytical challenges. Actually, the complexity–stability interplay of different ecological communities with realistic time delays remained unknown, though such knowledge can be important for ecosystem management and preservation. A comprehensive analytic investigation in this direction is lacking. The aim of our work is to address these pressing questions through dynamical systems theories to derive admissible complexity for different types of ecosystems and time delays together with some realistic considerations. Our main result is that, time delays reorder the previously established hierarchical order of admissible complexity. The stability–complexity trade-off is a fundamental ecological issue. Our findings indicate that conclusions drawn without considering realistic factors can be misleading.

## Results

### Complexity, time delay, and correlated interactions

To unveil the effect of time-delayed interactions on the interplay between the stability and complexity of ecosystems, we consider the following continuous-time system that describes the evolution of the abundances of *S* interacting species (21)


(1) $${\dot{y}}_{i}(t)=f(y(t),y(t-\tau )),$$

where $y(t)=[y_{1}(t),y_{2}(t),\ldots ,y_{S}(t){]}^{⊤}$ includes the abundance of species *i* to *S*, f(⋅,⋅) is smooth function, delineating the interactions, and τ⩾0 is a time delay. As we want to explore the local asymptotic stability of a strictly positive equilibrium $y^{*}$, we restrict our focus on the following linearized equation (28)


(2) $${\dot{x}}_{i}(t)=-dx_{i}(t)+\sum_{\;j=1}^{S}a_{ij}x_{\;j}(t-\tau ),\;i=1,\ldots ,S,$$

where $x_{i}(t)\;:=y_{i}(t)-y_{i}^{*}$ delineates the fluctuation of the *i*th species from its equilibrium abundance, and $a_{ij}$ characterizes the impact that species *j* has on *i* constituting a community matrix A. Those impacts are instantaneous when τ=0 and delayed ones for τ>0. Moreover, all species share the same strength of instantaneous self-interaction −d<0. We note that, according to the recent work (21), this is the case where self-interaction matrix −dI is commute with A. In addition, such stabilizing interactions can also be noninstantaneous. Here, we do not incorporate any assumption on the diagonal terms of the community matrix **A** for simplicity.

**Fig. 1.**
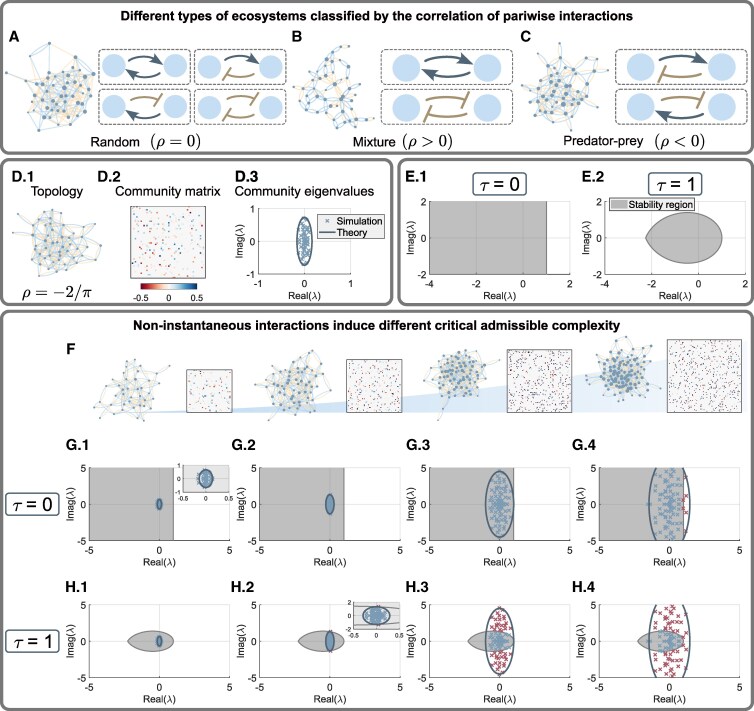
Delayed interactions change the critical admissible complexity of the predator–prey communities. A) An ecosystem with fully random community and its possible pairwise interactions (arrow: activation; T-shape: inhibition). Node size corresponds to the strength of total regulation it experiences. B) A mixed community includes two types of pairwise interaction: mutualism or competition. C) A predator–prey community. D.1) The interaction topology of a predator–prey community. D.2) The corresponding community matrix A with color-coded weights as defined by the color bar. D.3) All the eigenvalues of A (crosses) and the predicted distribution (solid ellipse). E.1) The stability region (gray) determined by the characteristic equation associated with Eq. 2 for τ=0. E.2) The stability region for τ=1. F) Four predator–prey communities with increasing complexity (from left to right), with the respective parameter values (S,σ,C)=(40,0.2,0.1), (60,0.35,0.0879), (80,1.1,0.0782), and (100,1.8,0.0494). G) For τ=0, the eigenvalues of the community matrices (crosses), corresponding to the ecosystems in (F), together with the stability region. Solid ellipses are theoretical prediction of the distribution of the community eigenvalues. G.3) This panel corresponds to the predicted critical admissible complexity $\alpha^{*}=2.7519$. In G.4), there are some eigenvalues (red crosses) outside the stability region. H) For τ=1, the eigenvalues of the community matrices (crosses: simulation; solid ellipses: theory) together with the stability region. H.2) The case associated with the critical admissible complexity $\alpha^{*}=0.8040$. In H.3) and H.4), there are eigenvalues (red crosses) outside the stability region is shown. The self-interaction d=1 is used for all panels.

Following previous works (2, 3), the community matrix has a sparsity parameter *C*, and every nonzero $a_{ij}$ is assigned randomly from a given distribution with zero mean and variance *σ*. The complexity of an ecosystem then becomes $\alpha =\sigma \sqrt{SC}$. For a particular ecosystem, there is also a pairwise correlation *ρ* describing the type of community (Methods). May’s original model assumed fully random communities (Fig. 1A) and established a stability bound with the critical admissible complexity $\alpha^{*}=1$ under unit self-interaction and the absence of time delay. In such a case, the maximal value of the sparsity parameter *C* is scaled as 1/S. Here, we regard them as two independent parameters. The model was later extended to other types of ecosystems including the mixed community (mixture) whose interaction pairs are either mutualistic or competitive (Fig. 1B) and the predator–prey one with direct-negative-feedback pairs (Fig. 1C). It was revealed mathematically that different types of delay-free ecosystems have different admissible complexity, and therefore, there is a strict hierarchical order of complexity (3). Particularly, the mixed communities have the least admissible complexity, followed by the random one, and the predator–prey communities accommodate the highest complexity. Because time delay was not considered, it is not clear whether more realistic ecological communities in nature would follow such a hierarchy.

### Discrete time delay changes the admissible complexity

#### Time delay mitigates the admissible complexity of the predator–prey systems

Throughout this study, we assess the stability of a given community in a binary sense. Particularly, the community is either stable or unstable when its complexity changes. The critical admissible complexity is the place where the transition occurs. This binary measure is different from the previously studied one where the recovery rate of a community is considered (27), and thus, we can now discuss how time delay affects the admissible complexity. Mathematically, local asymptotic stability is guaranteed when all eigenvalues *z* of the characteristic equation have negative real part. This criteria is equivalent to examining the position of the eigenvalues *λ* of the community matrix (Fig. 1D) and the stability region of the ecosystem (Fig. 1E, Methods). Specifically, the community is stable if all *λ* lie inside the stability region.

When time delay is introduced, the stability region changes from an open region (τ=0) to a closed leaf-shaped one (τ>0). A recent work showed that the recovery rate of the ecological community is altered by time-delayed effects (27). Here, we found that introducing time delay also changes the critical admissible complexity of the predator–prey community whose eigenvalues *λ* are distributed in a vertically stretched ellipse (Fig. 1D). The size of the ellipse grows monotonically as the complexity of the community increases. After introducing time delay, the stability region shrinks (Methods), the critical admissible complexity is then changed because some eigenvalues stay outside the region in the vertical direction (Fig. 1F–H). Therefore, the admissible complexity of the predator–prey community is mitigated by time-delayed effects. From a viewpoint of ecology, when τ>0, there is a phase shift on the effect of the direct-negative-feedback loop. If the abundance of species increases (or decreases), it is not suppressed (promoted) immediately but needs a period of time lag. Consequently, the time-delayed effects make the abundances fluctuate around their equilibrium, and thus, yield an unstable community.

#### The hierarchical complexity is reordered by time delay

**Fig. 2.**
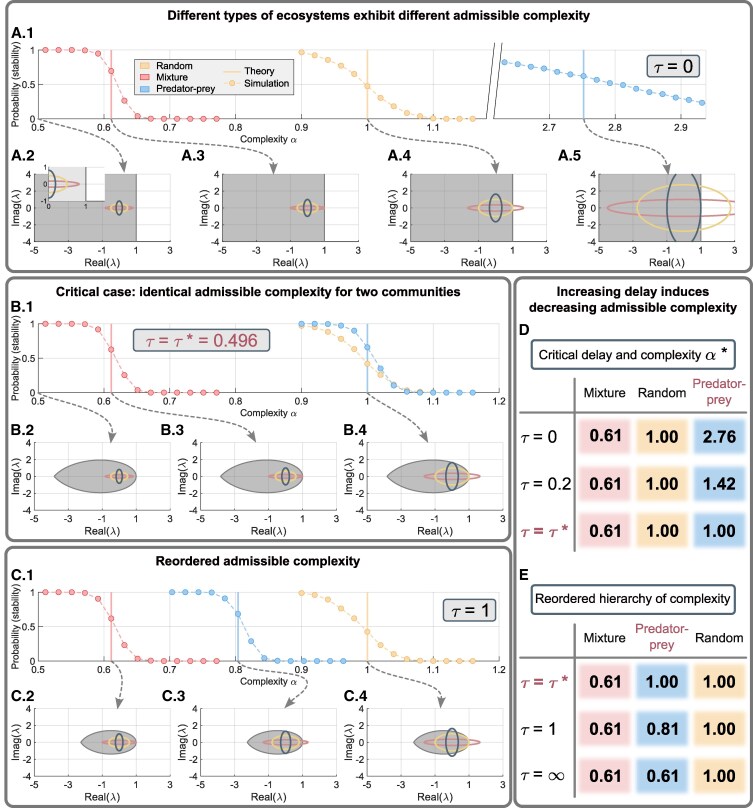
Reordered hierarchical complexity of three representative ecosystems. A.1) Probability of stability for three ecological communities (red: mutualism and competition; yellow: fully random; blue: predator–prey). Dots and dashed curves show the numerical results obtained from 100 runs for each complexity value *α*. The vertical lines indicate the predicted critical admissible complexity $\alpha^{*}$. A.2–A.5) Theoretical distribution of the eigenvalues of A for the three communities (circle and ellipses), where the stability region (gray) is also indicated. The panels from left to right correspond to α=0.5 and the three ordered critical admissible complexity, as indicated by the dashed arrows. For each critical case, the associated circle or ellipse is tangent to the vertical line ℜ(λ)=1. B.1) The probability of the three ecosystems for $\tau =\tau^{*}=0.0496$, where the predicted critical admissible complexity of the fully random and predator–prey communities are identical. B.2–B.4) Three distributions of the eigenvalues of A together with the stability region (gray) for different complexity *α*. In B.4), the distributions of fully random and predator–prey communities are both tangent to the boundary of the stability region (Methods). C.1) The probability of the three ecosystems for τ=1, where the critical admissible complexity of the predator–prey community is less than that of the fully random one. C.2–C.4) Three distributions of the eigenvalues of A together with the stability region (gray) for the reordered critical admissible complexity. D) Critical admissible complexity of the predator–prey community (blue) decreases as *τ* increases and becomes the same as that of the fully random one (yellow) for $\tau =\tau^{*}$. E) As *τ* increases further, the hierarchy of complexity for the three ecosystems are reordered. Other parameters are d=1, S=200, and C=0.2.

In the absence of time delay, predator–prey communities possess the greatest admissible complexity compared with fully random and mixed ones (Fig. 2A). Does the reduced admissible complexity of the predator–prey community induced by time delay change the hierarchical order? To address this question, we also analyzed the critical admissible complexity of the fully random and mixed communities. The difference among the three types of communities is the pairwise correlation *ρ* of the community matrix. It makes the distributions of the eigenvalues *λ* different (Methods). The eigenvalues of the fully random and the mixed community matrix are distributed respectively in a circle and a horizontally stretched ellipse (Fig. 2A).

It was already shown that the recovery rates of the fully random and the mixed communities are altered by time-delayed effects (27). Our analysis revealed a quite different conclusion that introducing time delay does not alter the critical admissible complexity of both communities, because the stability measure that we consider is a binary one. The mathematical underpinning is that the monotonically decreasing stability region yielded by time delay changes greatly in the vertical direction, and therefore, it does not affect the stability criterion in the horizontal direction (Fig. 2B and C). The conclusion can also be explained intuitively from a viewpoint of ecology. We note that both fully random and mixed communities have mutualistic or competitive interactions. For those pairs, though there is a phase shift, the effect of mitigation or promotion on the abundance of species remains consistent with the delay-free one. Consequently, those influences of direct-positive-feedback loops keep the same. The consequence of any perturbation yielding extinction or explosion of the fully random and mixed communities thus does not depend on the presence of time delay. We remark that the conclusion is valid when there is no more consideration on the community matrix A. If there are some assumptions like incorporating delayed self-interactions (i.e. restriction on the diagonal elements of A), the results may be changed.

Analyzing the critical complexity for the three types of ecological communities leads us to an interesting result. Introducing time delay breaks down the previously found hierarchy (3) and a new order emerges when the amount of time delay exceeds a threshold $\tau^{*}$ (Fig. 2B and C): fully random communities allow the greatest admissible complexity but the predator–prey ones belong to the middle and is never less than the mixed community for even longer delays (Fig. 2D and E). The implication is that, because of the ubiquity of time delay in nature, there is a significantly reduced chance that purely predator–prey communities with large community size or interactions could exist. Our result also suggests that the communities with mutualistic and competitive interactions are the most vulnerable ones among the three representative ecosystems.

#### The effect of positive and negative feedback loops

**Fig. 3.**
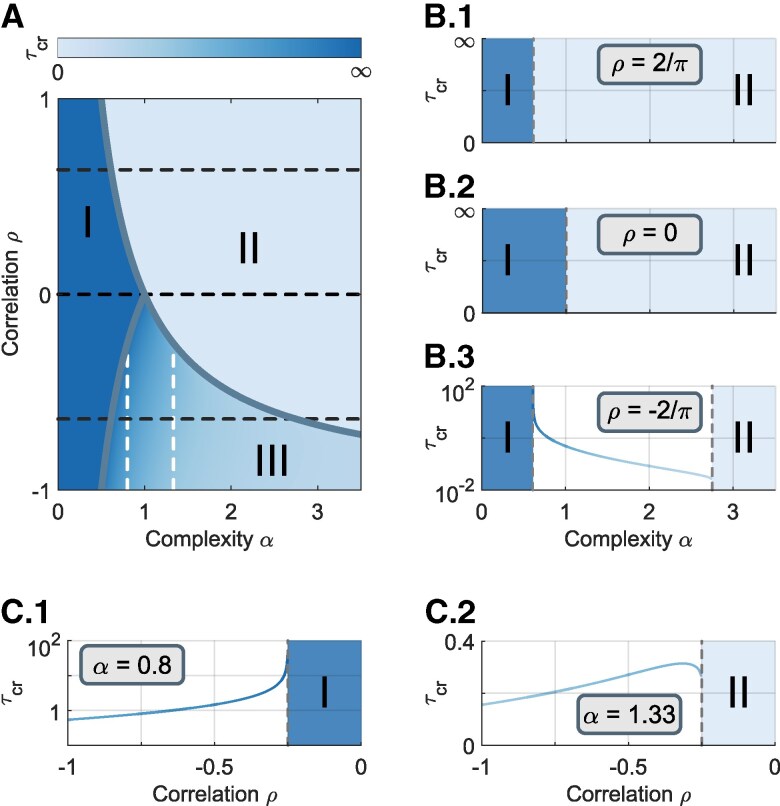
Critical time delay for instability. A) Heat map of the critical time delay $\tau_{\mathrm{cr}}$ for different correlation *ρ* and complexity *α*. Ecosystems in regions I and II, defined by $\tau_{\mathrm{cr}}=\infty$ and 0, respectively, are always stable and unstable. In region III, there is a color-coded critical value $\tau_{\mathrm{cr}}$ determined by *ρ* and *α*, below which the system is stable. B.1–B.3) Cross sections of the heat map for ρ=2/π, 0, and −2/π, respectively, as indicated by the three horizontal dashed lines. C.1, C.2) Cross sections of the heat map for α=0.8 and 1.33, corresponding to the two vertical lines (white) in (a).

Our results so far have indicated that an increasing time delay can make an originally stable predator–prey community unstable at a critical time delay $\tau_{\mathrm{cr}}$, while the stability of the other two types of communities remain unchanged. This is caused by different effects of negative- and positive-feedback loops. We now demonstrate an overview picture in Fig. 3 to see how the strength of those feedback loops influence the results, where the sign of *ρ* determines the type of community and its magnitude delineates the strength of feedback loops.

The diagram is divided into three regions. The community in regions I and II is respectively always stable or unstable regardless of the time delay, while in region III, the stability depends on the amount of time delay (color coded). The time delay affects the stability only when direct-negative-feedback loops involve (Fig. 3B). Additionally, the predator–prey communities with larger complexity are vulnerable to time-delayed effects. For the mixed communities (ρ>0), the admissible complexity decreases as the strength of positive-feedback loops increases. Analogous phenomenon is also observed for the predator–prey community (ρ<0). Moreover, for a particular complexity, as the strength of negative-feedback loops increasing, the community losses its stability at a lower amount of time delay (Fig. 3C). Those conclusion inform us that the stability can be hardly maintained when the type of pairwise interactions in the community becomes extreme (|ρ|→1).

### Distributed time delays induce nonmonotonicity

**Fig. 4.**
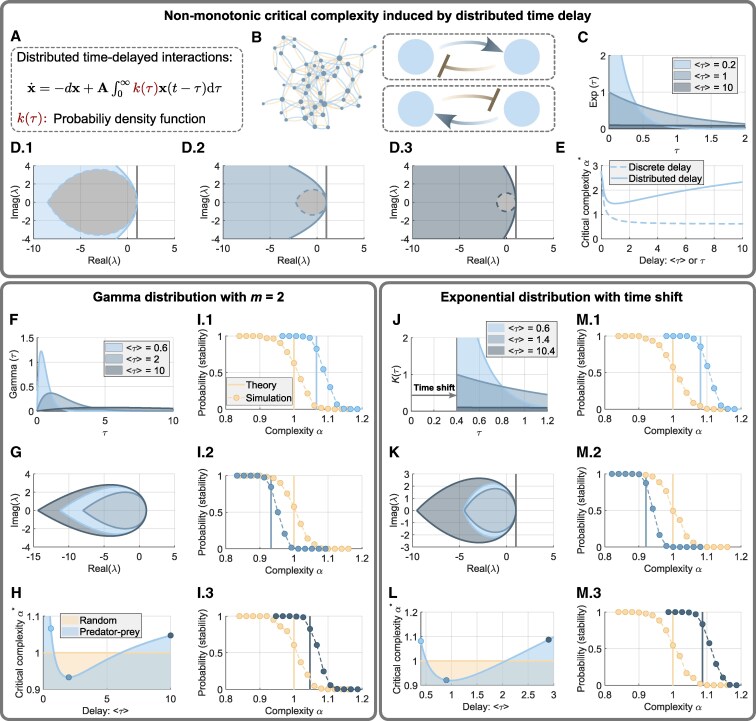
Nonmonotonic and reordered critical admissible complexity induced by continuous time delays. A) A linearized ecosystem with continuously time-delayed interactions characterized by the weighted (kernel) function k(τ). B) A predator–prey community with continuous time delay (gradient color). C) Three delay kernels: exponential distributions correspond to different means. D.1–D.3) Stability region obtained from the characteristic equation of Eq. 3 (colored region on the left of the solid blue curves) with the three distributions given in (C). The gray area bounded by the dashed curves are stability regions associated with the discrete time delay τ=⟨τ⟩. E) Theoretical critical admissible complexity $\alpha^{*}$ of the predator–prey community with distributed (solid) and discrete (dashed) time-delayed interactions. F) Three Gamma distributions with different means. G) The area bounded by middle, inner, and outer closed curves indicating the stability regions associated with the distributions in (F) with ⟨τ⟩=0.6, 2, and 10, respectively. H) Critical complexity $\alpha^{*}$ of the fully random (yellow) and predator–prey (blue) ecosystems when the kernel function is a Gamma distribution with different mean ⟨τ⟩. I.1–I.3) Probability of stability (dots: simulation; vertical lines: theory) of the two ecosystems highlighted by the circles in (H). As the average delay ⟨τ⟩ increases, the hierarchy of complexity is reordered (I.2) and then restored (I.3). J) Three exponential distributions with a constant time shift $\hat{\tau }=0.4$. K) Three stability regions corresponding to the three distributions in (J). L) Critical complexity versus the average delay ⟨τ⟩ of a time-shifted exponential distribution. M.1–M.3) Probability of stability of the ecosystems considered and highlighted in (L). The hierarchy of complexity is reordered and then restored as average delay increases.

In practice, discrete time delay may not fully characterize the delayed effects among the species. Actually, the “waiting time” or the occurrence of an action (e.g. predation) can be better described by a statistical distribution k(τ) (21) (Methods). Then, the evolution of the abundance depends on the past over a continuous time period (11–13) yielding the following linearized model (see Fig. 4A for the matrix form)


(3) $${\dot{x}}_{i}(t)=-dx_{i}(t)+\sum_{\;j=1}^{S}a_{ij}\int_{0}^{\infty }k(\tau )x_{\;j}(t-\tau )d\tau .$$

Intuitively, distributed delays are effectively normalized weighted time delays.

We studied analytically the representative distributed delays where k(τ) is the Gamma function (Methods). The only difference between discrete and distributed delays is the size of the stability region. For discrete one, the region shrinks monotonically as the time delay increases. But, we surprisingly found that the region changes in a nonmonotonic behavior for the distributed case as the average delay ⟨τ⟩ increases (Fig. 4C and D, Methods). It first shrinks and then expands. Accordingly, the critical admissible complexity of the predator–prey community depends also nonmonotonically on the average delay (Fig. 4E). Intuitively, we may regard the delay effect as an “ecological memory.” As the average “memory” increases slightly, its effect becomes stronger and thus the tendency is analogous to a discrete one. Further increasing the average delay akin to putting the “memory” at every moment in the past but with very little amount. The consequence is however like the ecosystem forgetting everything happened before.

A direct and interesting consequence of the distributed delays is that the variation in the hierarchical order of communities’ complexity is also nonmonotonic. We considered three Gamma distributions with different average (Fig. 4F). As expected, when the average delay increases, the stability region and the critical complexity of the predator–prey community changes nonmonotonically (Fig. 4G and H). We also showed that the stability of the fully random and the mixed communities are not altered by the distributed delays (Methods). Therefore, as average delay increases, the hierarchical order alters twice (Fig. 4H). Compared with the delay-free case, the hierarchy is reordered with a moderate average delay, but eventually recovers (Fig. 4I). Such a behavior is characteristically different from that with a discrete-time delay, where the hierarchical order cannot be recovered once the critical threshold $\tau^{*}$ is attained. The change and the recovery of the hierarchical order require two conditions: (i) the distributed delays induce a nonmonotonic critical complexity for the predator–prey community and (ii) its least critical complexity should be less than that of the fully random community (Fig. 4H).

We also studied another practical case when there is a time shift (29) $\hat{\tau }$ of the distributed delay (Fig. 4J and K). It is regarded as an inherent delay and has been incorporated into the analysis of insect population dynamics (29, 30). The inherent delay actually strengthens the overall delay effect (Fig. 4E and L). It therefore makes the least critical complexity of the predator–prey community less than that of the fully random one. Consequently, the reordering of the hierarchical complexity and restoration can be anticipated and observed (Fig. 4M).

### Further considerations of realistic significance

**Fig. 5.**
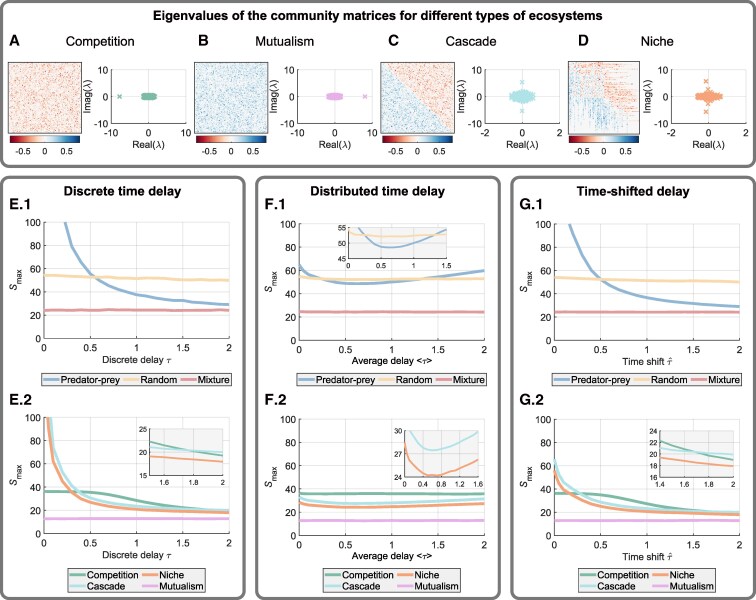
Admissible capacities $S_{\max}$ for various representative ecosystems. A) The community matrix **A** of an ecosystem with solely competitive interactions, i.e. $a_{ij}<0$. Right panel shows its eigenvalues. B) The ecosystem with solely mutualistic interactions: $a_{ij}>0$. C) A predator–prey community manifested as a cascading food chain. D) A predator–prey community with a niche food-web structure. E) Numerically obtained admissible capacity $S_{\max}$ for the seven representative ecosystems (red: mixture; yellow: fully random; blue:predator–prey; dark green: fully competition; purple: fully mutualism; light green: cascade; orange: niche) with discrete time delay. F) Admissible capacity for the seven ecosystems with continuous time delays with varying average delay. Time delays are characterized by an exponential distribution with mean ⟨τ⟩ together with a fixed time shift $\hat{\tau }=0.4$. G) Admissible capacity for the seven ecosystems with time-delayed interactions, where the time delays are characterized by an exponential distribution with fixed mean ⟨τ⟩=0.1 together with a varying time shift $\hat{\tau }$. Other parameters are d=1, C=0.5, and σ=0.2.

The analysis we have performed so far is based on the theory of random matrices, which require sufficiently large community size *S*. Does the reordering phenomenon persist when *S* is not large, e.g. S<100? Also, there are more possible structures of communities in natural ecosystems. Here, we introduce four more ecosystems (Fig. 5A–D), to test the generality of our analytical results. They are classified into two types (Sections 5.1–5.2 in SI). (i) The first two contain purely competitive and mutualistic effects (31) (Figs. 5A and B). (ii) The other two communities are of the predator–prey type following certain food-web structures including the cascading one (32) and the niche one (33, 34) (Figs. 5C and D). Altogether we studied seven types of ecosystems.

Because we now focus on validating our theoretical results for relatively small community size, we use the maximal capacity $S_{\max}$ of each ecosystem to characterize the critical complexity provided that the other two parameters *σ* and *C* are fixed. We computed the maximal capacity $S_{\max}$ of those ecosystems indicating the critical complexity of the system (Sections 3.2–3.3 in SI). The cases of discrete time delay, distributed delays with varying average and distributed ones with varying time shift were investigated and their results are shown respectively in Fig. 5E, F, and G. Despite the small community size, tendencies of variation in the maximal capacity analogous to those for large communities arise. The mixed and fully random communities exhibit near-constant maximal capacity in all cases. The capacity of the predator–prey community decreases significantly and tends to the mixed one as increasing the discrete and shifted time delay, yielding the reordering of hierarchy. The nonmonotonicity induced by the distributed delays is also observed producing the reordering and restoration of the hierarchy.

Considering the additional communities, the results for discrete and time-shifted delays are analogous (Fig. 5E.2 and G.2). The purely mutualistic and competitive ecosystems possess near-constant capacity for small delays–analogous to the mixed one. However, for the competitive one, the capacity decreases as time delay approaches a certain value because the capacity is determined by whether the left-most eigenvalue of the community matrix lies inside the stability region (Section 5.1 in SI). The capacities of the two communities with realistic food-web structures are further suppressed, compared with the (random) predator–prey one. They become even smaller than the capacity of the mixed community. This is consistent with the previous result that realistic food-web structures hamper the stability of such ecosystems (3). The mitigation of the capacity together with the nonmonotonicity is also observed when distributed time delays with an increasing average are considered (Fig. 5F.2). In this case, the capacities of purely mutualistic and competitive ecosystems remain almost static. Those simulations indicate the absolute necessity to take into account time delays in forecasting the maximal complexity of realistic ecosystems. Moreover, our theoretical results can also be used to understand the behavior of those communities with small size.

The community matrix A is a significant factor for determining the critical complexity. Its nonzero elements follow a given distribution. We consider whether and how the distribution, from which the entries of the community matrix A are drawn, influences the critical complexity. According to the elliptic law (35), for large random matrices, the distribution of eigenvalues depends solely on the mean, variance, and correlation *ρ* of the matrix entries, regardless of the specific form (e.g. shape) of the distribution. In the case of random communities (ρ=0), this implies that different distributions (e.g. Gaussian and uniform) with identical mean and variance yield the same distribution of eigenvalues and thus result in the same critical complexity.

When considering structured communities (ρ≠0), such as the type of predator–prey and mixture, we note that different distributions—with the same mean and variance—can induce different correlations *ρ* between matrix entries. According to the elliptic law (35), these variations in *ρ* lead to difference in the distributions of eigenvalues, which further affect the critical complexity. The influence of distributions with different correlations is analyzed in detail in Section 4.2 of SI.

In particular, we studied the predator–prey community and examined how the correlation *ρ*, together with time delay, affects the critical complexity. We found that when the delay is small, increasing |ρ| raises the corresponding critical complexity, whereas when the delay is large, increasing |ρ| lowers it. Our results are consistent with the elliptic law and extend the conclusions of previous work (3). The results also highlight the complex interplay between delay and correlation in shaping the stability of structured communities.

We consider two more factors that could occur naturally. (i) First, pairwise effects could have heterogeneous time delays. We considered a perturbation to the time delay (Section 4.1 in SI) and showed that the reordered hierarchy is maintained even with considerable perturbation. (ii) Second, we considered asymmetric time delays for three predator–prey type communities. Time delays are only introduced to those effects from preys to predators. We observed qualitatively the same results as those with bidirectional time delays for relatively small delay values (Section 5.3 in SI), indicating that the reordering of hierarchy is robust and can be anticipated in complex ecosystems.

Finally, we addressed the issue of the length of time delay. We showed that it is inversely proportional to the growth rate of the concerned species. For example, if the growth rate is 10/year, the time delay would be on the order of a month (Section 6 in SI). The value is relevant to natural species such as insects, mammals, and birds (30). These data indicate that the amount of time delays considered here are reasonable, providing insights into their effect on the hierarchical complexity of ecosystems in the real world.

## Discussion

The possible complexity or capacity of large, complex ecosystems is of paramount significance to the sustainability of life on Earth and in particular the human society. We have performed a comprehensive analysis with rigorous mathematical reasoning to assess the admissible complexity of representative types of communities with noninstantaneous interactions. When time delays are present, to keep stable abundances, any ecosystem can only accommodate a limited community size with a certain number or uncertainty of interactions. While our result is consistent with previous ones (2, 3), we additionally uncover that any type of time delays encompassing discrete, distributed and time-shifted ones affect the admissible complexity of predator–prey type of ecosystems dramatically as the amount of delays changes. Contrarily, fully random communities and those with mutualistic or competitive interactions are hardly influenced. These findings suggest that the previously established complexity hierarchy for different types of delay-free ecological communities (3), which is one of the fundamental issues in contemporary ecology, needs to be reexamined.

Indeed, our analysis revealed a reordering of the hierarchical complexity when incorporating time delays. As the time delay (discrete and time-shifted) increases, the complexity of the predator–prey community is compromised because of the effect of direct-negative-feedback loops. This striking finding indicates that, in sharp contrast to the previous result (3), large and complex predator–prey communities are generally unstable in the presence of time delays. One possible implication is that, because of the ubiquitous presence of time delay in natural ecosystems, it is unlikely to observe large, complex uncertain predator–prey type of ecosystems. From an alternative viewpoint, time delays can be an important contributing factor to diversity in nature due to the richer dynamical behaviors that can arise in such complex networked systems.

Recently, discrete time delays have been shown to have the ability to modulate the recovery rate of many types of ecosystems with certain complexity (27). Our work focuses on a different measure, the ecological complexity, an issue that has not been addressed before. Different from previous result, we showed that time delays only alter the complexity of predator–prey type of communities significantly, which is the major reason for the reordered hierarchy. In addition to ecological findings, our rigorous analysis of the stability bound of ecosystems with distinct types of community matrix and different types of time delays provides a solid foundation for understanding the interplay between ecological stability and complexity in nature. Technically, our analysis enables us to calculate the exact critical admissible complexity $\alpha^{*}$ for a given time delay *τ*, which is mathematically quite challenging. We note that this critical value was only estimated in the recent work (27).

Further, we also provided analytic results when ecosystems have continuous “memory” (distributed time delays). The most important finding is possibly the nonmonotonic variation in the complexity of the predator–prey communities with respect to the average of the continuous delays. As a comparison, the critical complexity of the random and mixed communities are unchanged. Therefore, while the hierarchical complexity is reordered as the overall amount of time delay increases, the order can finally be restored when the amount exceeds a certain threshold. This result is important for understanding complex ecosystems, as it can guide us to choose the correct dynamical models to analyze ecological memories. It is worth pointing out that the nonmonotonic variation may not occur by changing other moments of the distribution (e.g. changing variance with a fixed average). We also established the generality of our analytic results, which were obtained for large systems, for more realistic ecosystems with small community sizes and other realistic factors.

Taken together, our work provides fresh insights into the fundamental interplay between stability and complexity in more realistic ecosystems. There are also future directions that can strengthen our understanding. As shown in a recent work (21), incorporating noninstantaneous self-interaction alters stability pattern significantly and can also induce nonmonotonic phenomenon for the random communities. This raises significant questions about how delayed self-interactions may affect the critical complexity of various community types and their hierarchical order, which deserve further exploration. Also, we focus on the local asymptotic stability in the present work which characterizes the dynamics only in the vicinity of the equilibrium. It thus provides limited information on the stability of complex ecosystems. To have a more comprehensive understanding, other significant metrics including reactivity (36) and recovery time (27) merit further detailed investigation. Finally, there are inherent limitations of the linearized model. The relationship between complexity and stability in generic ecological models as formulated in Eq. 1 remains a formidable challenge that requires innovative analytical and computational approaches.

## Methods

### Community matrix and correlated interactions

The parameters $a_{ij}$ in Eq. 2 constitute a community matrix $A\;:\;=\{a_{ij}{\}}_{i,j=1}^{S}$. The sparsity of the community is characterized by the probability of the existence of each interaction $a_{ij}$: C∈(0,1]. Each nonzero element is then randomly generated from a give distribution with zero mean and variance *σ*. See Sections 3 and 5 in SI for the details on the configuration of community matrices for different types of ecosystems.

Throughout this study, we use the correlation parameter, defined as $\rho \;:\;=\langle a_{ij}a_{\;ji}\rangle$, to characterize the pairwise relationship among species (Section 2.1 in SI). It is used to classify and distinguish the network interaction patterns. For ρ=0, the community matrix becomes identical to that in May’s original model, where the structure is homogeneously random (Fig. 1A). For ρ>0, there is a tendency for any two species in the ecosystem to simultaneously promote or suppress each other’s abundances (Fig. 1B). For ρ<0, the pair of species tends to exhibit opposite behaviors (Fig. 1C). An ecosystem with ρ>0 thus corresponds to a community with mixed competitive and mutualistic interactions, while ρ=0 represents a random community, and ρ<0 is characteristic of predator–prey ecosystems (3). The magnitude of *ρ* represents the level of congruity of pairwise interactions and can be used to characterize the strength of direct-feedback loops.

### Distribution of eigenvalues of the community matrix

According to the *elliptic law* (37), the distribution of the eigenvalues *λ* of the community matrix A depends only on the complexity $\alpha =\sigma \sqrt{SC}$ and the correlation *ρ* (Theorem 2 in SI). In particular, for ρ=0, ρ<0, or ρ>0, the eigenvalues *λ* are distributed uniformly within a circle $\Omega_{A}^{c}$, a vertically stretched ellipse $\Omega_{A}^{v}$, or a horizontally stretched ellipse $\Omega_{A}^{h}$ in the complex plane, respectively. An example of a predator–prey system is shown in Fig. 1D.3.

### Stability region affected by time delay

For an ecological community with a large community size *S*, determining its critical admissible complexity requires the relationship between the distribution of the eigenvalues of the community matrix A and the stability region calculated from the associated characteristic equation which is influenced by the time delay (Section 2 in SI). Figure 1E shows examples of the stability regions (shaded area) when τ=0 and τ=1. When all the eigenvalues of the community matrix A lie in the stability region, the corresponding complexity *α* is admissible, guaranteeing the stability of the equilibrium abundances.

For delay-free ecosystem, the stability region is the open left plan with ℜ(λ)<d. The ecosystem possesses stable equilibrium abundances if the real parts of all eigenvalues of A are less than *d*. When a discrete time delay is present (0<τ<+∞), the stability region becomes a leaf-shaped area, denoted by $\Omega_{\tau }$ (see Section 2.1 in SI for a detailed analysis). A larger time delay leads to a smaller leaf-shaped stability region $\Omega_{\tau }$ and, for τ→∞, $\Omega_{\tau }$ approaches $\Omega_{\infty }$–a disk of radius *d* centered at the origin (see Fig. S1 in SI).

### Finding the critical admissible complexity

The locations of $\Omega_{A}^{c,v,h}$ and $\Omega_{\tau }$ enable the critical complexity $\alpha^{*}$ to be calculated in terms of the correlation *ρ* and the time delay *τ*. Because the stability region shrinks monotonically to $\Omega_{\infty }$, there is a disk centered at the origin with radius *d* (Section 2.1 in SI). For τ→+∞, the distributions $\Omega_{A}^{c}$ and $\Omega_{A}^{h}$ are bounded by $\Omega_{\tau }$ when their rightmost points do not cross (d,0). Consequently, the critical admissible complexity of the fully random (ρ=0) and mixed (ρ>0) communities correspond respectively to the cases where $\Omega_{A}^{c}$ and $\Omega_{A}^{h}$ are tangent to the stability region at (d,0) (Fig. 2B.3 and B.4). The critical complexity can then be inferred as $\alpha^{*}=d/(1+\rho )$ for ρ≥0, which depends on *ρ* and *d* but not on *τ*. This result implies that May’s stability bound (2) for random communities (ρ=0) without time delay is valid for determining the critical admissible complexity even in discrete delayed ecosystems with ρ≥0. Alternatively, for ρ<0, admissible complexity requires that the vertical extension of $\Omega_{A}^{v}$ is bounded by $\Omega_{\tau }$ (Fig. 1H). In this case, the critical complexity $\alpha^{*}$ relies on the values of both *ρ* and *τ*, which can be obtained by solving the following two algebraic equations


(4) $$\begin{array}{cc} F_{1}(\rho ,\tau ,\omega ,\alpha ) & =\;0,\\ F_{2}(\rho ,\tau ,\omega ) & =\;0. \end{array}$$

The explicit expressions of $F_{1}$ and $F_{2}$ are given in Section 2.1 of the SI. They are obtained by considering the critical case when the distribution of the eigenvalues of A and the stability region intersect with one another (see Section 2.1 of the SI for more details). For a set of fixed *ρ* and *τ*, the unknowns $\alpha^{*}$ and $\omega^{*}$ are solved. Here, $\omega^{*}$ is the critical frequency corresponding to the unstable eigenvalues.

As exemplified in Fig. 2a for τ=0, our analysis enables the critical admissible complexity of the three representative ecosystems to be predicted. The results are also verified by simulations, as in Fig. 2A.1, where each dot indicates the numerical probability of stable ecosystems.

### The complexity of the predator–prey community

A direct consequence of a discrete time delay is a decreasing stability region. It is reasonable to expect that a time delay will change the critical (greatest) admissible complexity of a predator–prey community, whose eigenvalues are distributed in a vertically stretched ellipse $\Omega_{A}^{v}$. This is verified numerically in Fig. 1F–H. As the complexity of the predator–prey system increases (Fig. 1F), the corresponding eigenvalue distribution is enlarged. When there is no time delay, the critical admissible complexity is such that the distribution of the eigenvalues is tangent to the vertical line Re(λ)=d (Fig. 1G.3). For τ=1, the critical case occurs earlier in the sense that the boundaries of $\Omega_{A}^{v}$ and the stability region become tangent to each other at a point with less complexity (Fig. 1H.2), due to the tangent point occurring in the vertical direction. More insights can be gained by analyzing the limiting case of τ→+∞. Our results show that the critical admissible complexity of the predator–prey systems cannot be less than that of the mixed ones (Fig. 2E).

### Critical time delay

As mentioned previously, we are able to find accurately the critical admissible complexity $\alpha^{*}$ when *ρ* and *τ* are given. Sometimes, it is also necessary to find the critical time delay $\tau_{\mathrm{cr}}$, where the ecosystem with certain complexity loses its stability. For instance, at a threshold $\tau_{\mathrm{cr}}$, the predator–prey communities share the same critical complexity $\alpha^{*}=d$ as that of the fully random ones (Fig. 2B). The value of $\tau_{\mathrm{cr}}$ and the corresponding critical frequency $\omega^{*}$ can also be computed from the algebraic Eq. 4 (see Section 2.1 in SI) by fixing *ρ* and $\alpha^{*}=d$.

### Continuous time delay

We use Eq. 3 to model the linearized dynamics of ecosystems with continuous time delay. The function k(τ) is called the kernel function satisfying the practical conditions k(τ)≥0 (nonnegativity) and $\int_{0}^{\infty }k_{0}(\tau )\;d\tau =1$ (normalization). The average delay is calculated as $\langle \tau \rangle \;:\;=\int_{0}^{\infty }\tau k(\tau )\;d\tau$.

The Gamma function used in the main text is written as $k(\tau )=a^{m}\tau^{m-1}\exp\;(-a\tau )/\Gamma (m)$ with s>0, a>0, and m>0. For m=1, where k(τ) degenerates into an exponential distribution aexp(−aτ),τ>0, giving ⟨τ⟩=1/a. Analytically, the stability region can also be calculated from the characteristic equation as (Section 2.2 in SI)


(5) $$\Omega_{\exp}=\left\{\lambda =x+\text{i}y|x\leq d-\frac{a}{(a+d{)}^{2}}y^{2}\right\},$$

which describes an unbounded region (Fig. 4D.1–D.3) where, for comparison, the bounded stability region (gray area) for the case of discrete time delay, which shrinks monotonically with τ=1/a=⟨τ⟩, is also shown. The critical complexity $\alpha^{*}$ for the fully random and the mixed ecosystems is invariant as the average delay increases because the smallest stability region is larger than $\Omega_{\tau }$, the disk centered at the origin with radius *d*. More results and discussions on the continuous time delays can be found in Section 2.2 in SI.

### Time-shifted delay

When the ecosystem possesses an inherent time delay, the density function k(τ) is shifted by a positive time $\hat{\tau }$: $k(\tau )\;:\;=k_{0}(\tau -\hat{\tau })$ for $\tau >\hat{\tau }$, where $k_{0}(\tau )$ (τ>0) is a given density function satisfying $k_{0}(\tau )\geq 0$ (nonnegativity) and $\int_{0}^{\infty }k_{0}(\tau )\;d\tau =1$ (normalization). A detailed analysis of ecosystems with a shifted density function is provided in Section 2.2 in SI.

## Supplementary Material

pgaf214_Supplementary_Data

## Data Availability

All data are included in the manuscript and supporting information.
