# Supplementary material for: Reordered hierarchical complexity in ecosystems with delayed interactions
Source: PNAS Nexus. 2025 Jul 14;4(7):pgaf214. doi: 10.1093/pnasnexus/pgaf214 (PMC12280872; doi:10.1093/pnasnexus/pgaf214)
Supplement: pgaf214_Supplementary_Data [file pgaf214_supplementary_data.pdf]

Supplementary Information for

Reordered hierarchical complexity in ecosystems with delayed interactions

Bo-Wei Qin#, Wenbo Sheng#, Xuzhe Qian, Jürgen Kurths, Alan Hastings, Ying-Cheng Lai✉ & Wei Lin✉

#: B.-W.Q. and W.S. contributed equally to the work.

✉: Ying-Cheng.Lai@asu.edu; wlin@fudan.edu.cn

Contents

1 Preliminaries . . . **2**
2 Mathematical analyses on the critical admissible complexity . . . **3**
2.1 Ecosystems with discrete time delays . . . 3
2.1.1 Cases when $\rho \geqslant 0$ . . . 4
2.1.2 Cases when $\rho < 0$ . . . 4
2.2 Distributed time delays and time shift $\hat{\tau} \geqslant 0$ . . . 7
2.2.1 The Gamma distribution . . . 8
2.2.2 Some general results for the cases when $\rho \geqslant 0$ . . . 9
2.2.3 Some general results for the cases when $\rho < 0$ . . . 10
3 Detailed settings for numerical simulations . . . **13**
3.1 Configure the three representative types of ecosystems . . . 13
3.2 Estimating admissible capacity $S_{\max}$ . . . 13
3.3 Corresponding results for discrete time delays . . . 14
4 Effects of other realistic factors . . . **15**
4.1 Robustness of altered complexity hierarchy with respect to heterogeneous time delays . . . 15
4.2 Effects of different probability distributions for interaction strength on the stability hierarchy . . . 15
5 Extended ecosystems with more interacting structures . . . **17**
5.1 Competitive and mutualistic ecosystems . . . 17
5.2 Cascade and niche predator-prey ecosystems . . . 18
5.3 Effect of asymmetric delays in predator-prey type ecosystems . . . 19
6 Estimation of the amount of time delay . . . **20**
7 Supplementary table . . . **22**
8 Supplementary figures . . . **23**

1 Preliminaries

A central tool in the stability-complexity analysis of networked ecosystems is the spectrum theory of large random matrices that characterizes the distribution of the eigenvalues of the community matrix. In recent decades, there have been dramatic advances in the random matrix theory and it has now become possible to study the those properties of complex systems arising from a variety of fields [1, 2, 3, 4].

Regarding the fully random community, consider elements a_{ij} of matrices $\mathbf{A}$ drawn independently from given probability distribution. The distribution of eigenvalues λ was studied under different auxiliary conditions [5, 6] and the fundamental result is the theorem due to Tao *et* al. [7].

Theorem 1 (Circle Law)**.** *Let* $\mathbf{A}$ *be an* $S \times S$ *random matrix whose entries are independently identically distributed (i.i.d) random variables with zero mean and unit variance. The empirical distribution of eigenvalues of* $\mathbf{A}/\sqrt{S}$ *converges to a uniform distribution on the unit disk both in probabilistic and in the almost everywhere sense as* $S \to \infty$*.*

A more general case is that the ensembles are not independent but have correlation. For instance, in ecological systems, there can be statistical correlation between the pairwise elements a_{ij} and a_{ji}. Numerical simulations revealed that the behavior of the distribution of eigenvalues obeys an elliptic law [8, 9, 10, 11, 12]. A universal result about the elliptic law has been established [12], which can be stated, as follows.

Theorem 2 (Elliptic Law)**.** *Consider real matrices* $\mathbf{A} = \{a_{ij}\}_{i,j=1}^{S}$ *whose elements* a_{ij} *are drawn from given probability distribution and satisfy the following conditions:*

(i) the pairs $(a_{ij}, a_{ji}), i \neq j$ *and the diagonal elements* a_{ii} *are independent of each other;*

(ii) the values of mean, variance, and correlation satisfy $\mathbb{E}[a_{ij}] = 0$, $\mathtt{var}[a_{ij}] = 1$ *and* $\mathbb{E}[a_{ij}a_{ji}] = \rho$ *(if* $i \neq j$*) for some* $|\rho| \leq 1$*;*

(iii) a_{ij} *are uniformly square integrable, i.e.,* $\max_{i,j}\left\{\mathbb{E}\left[a_{ij}^2 \,\middle|\, |a_{ij}| > M\right]\right\} \to 0$*, as* $M \to +\infty$*.*

For $|\rho| < 1$*, the empirical distribution of the eigenvalues of* $\mathbf{A}/\sqrt{S}$ *converges weakly in the probabilistic sense to the uniform distribution on the ellipse* Ω *that depends on* ρ *as* $S \to \infty$*, where*

$$\Omega = \left\{\lambda = x + \mathrm{i}y \in \mathbb{C} \,\middle|\, \frac{x^2}{(1+\rho)^2} + \frac{y^2}{(1-\rho)^2} \leq 1\right\}.$$

Note that when $\rho = 1$ (i.e., symmetric $\mathbf{A}$) reduces the elliptic law to the Wigner's semi-circle law [13]. For $\rho = 0$, the elliptic law degenerates into the circle law.

In our study, we assume that the random matrices delineating the pairwise interactions among species satisfy the conditions required by the existing results as stated above. The procedure of generating certain community matrices is given in the latter section. Remark that the conclusions drawn from random matrix theory are valid to study the critical admissible complexity in our work when the community size S is assumed to be large enough. Actually, we also study the cases when S is relatively small by numerical simulations to validate the conclusions.

2 Mathematical analyses on the critical admissible complexity

In this section, we carry out mathematical analyses on the admissible complexity α for different types of ecosystems. The critical admissible complexity is the threshold where the equilibrium abundances of a given ecosystem change from stable to unstable. Therefore, we perform stability analysis for the linearized system with either discrete and or distributed (continuous) time delays.

2.1 Ecosystems with discrete time delays

As mentioned in the main text, the linearization of an ecosystem with discrete time delay around the equilibrium abundances is written as

$$\dot{\boldsymbol{x}}(t) = -d\boldsymbol{x}(t) + \mathbf{A}\boldsymbol{x}(t-\tau), \tag{S1}$$

where $\boldsymbol{x} = (x_1, \ldots, x_S)^\top$, S is the community size, and $\mathbf{A}$ is the community matrix of size $S \times S$. We consider matrices that satisfy the conditions of Theorem 2. Specifically, the elements a_{ij} of $\mathbf{A}$ are random variables satisfying

$$\mathbb{E}[a_{ij}] = 0, \quad \texttt{var}[a_{ij}] = C\sigma^2, \quad \mathbb{E}[a_{ij}a_{ji}] = \rho, \tag{S2}$$

where $\sigma > 0$, $-1 \leqslant \rho \leqslant 1$ and $C \in (0, 1]$. Moreover, the pairs (a_{ij}, a_{ji}) and the diagonal elements a_{ii} are independent of each other. As defined in the main text, the complexity of an ecosystem is $\alpha := \sigma\sqrt{SC}$.

We are now going to analyze the critical admissible complexity of the equilibrium $\boldsymbol{x} \equiv \mathbf{0}$ of Eq. (S1). As we will see soon, the value is related to the location of the eigenvalues λ of the community matrix $\mathbf{A}$. According to the elliptic law (Theorem 2), when the community size S is sufficiently large, the distribution of the eigenvalues approaches an uniform distribution on an ellipse

$$\Omega_{\mathbf{A}} = \left\{ \lambda = x + \mathrm{i}y \,\middle|\, \frac{x^2}{\alpha^2(1+\rho)^2} + \frac{y^2}{\alpha^2(1-\rho)^2} \leq 1 \right\}. \tag{S3}$$

We denote $\lambda_{\mathbf{A}}(\alpha, \rho)$ as the boundary of the ellipse which is significant for analyzing the critical admissible complexity. Remember that the critical admissible complexity is the threshold where the equilibrium loses stability.

We then decompose the community matrix as $\mathbf{A} = \mathbf{P}\mathbf{\Lambda}\mathbf{P}^{-1}$, where $\mathbf{\Lambda}$ is the Jordan form of $\mathbf{A}$ and $\mathbf{P}$ is the corresponding invertible $S \times S$ matrix. Under the linear transformation $\boldsymbol{x} = \mathbf{P}\boldsymbol{u}$ with $\boldsymbol{u} = (u_1, u_2, \cdots, u_S)^\top$, we obtain the following set of differential equations for scalar variables u_i, $i = 1, 2, \cdots, S$

$$\dot{u}_i(t) = -du_i(t) + \lambda_i u_i(t-\tau), \tag{S4}$$

where λ_i are the eigenvalues of $\mathbf{A}$.

For the sake of simplicity, we drop the subscript i hereafter and bear in mind that each eigenvalue of the community matrix $\mathbf{A}$ yields a differential equation in the form of Eq. (S4). Moreover, analyzing the stability of the equilibrium $\boldsymbol{x} \equiv \mathbf{0}$ of Eq. (S1) is equivalent to study the characteristic equation corresponding to Eq. (S4). Therefore, we leverage the ansatz $u(t) = \mathrm{e}^{zt}$, $z \in \mathbb{C}$ to obtain the following characteristic equation

$$H(z) := z + d - \lambda \mathrm{e}^{-z\tau} = 0. \tag{S5}$$

If all the roots of Eq. (S5) satisfy $\mathrm{Re}(z) < 0$, then the equilibrium abundance that we consider is stable. Therefore, we insert the critical condition $z = \mathrm{i}\omega$, $\omega \in \mathbb{R}$ into Eq. (S5) and obtain

$$\hat{H}(\omega) := H(\mathrm{i}\omega) = \mathrm{i}\omega + d - \lambda \mathrm{e}^{-\mathrm{i}\omega\tau} = 0.$$

Equivalently, we have $\lambda(\omega, \tau) = \mathrm{e}^{\mathrm{i}\tau\omega}(d + \mathrm{i}\omega)$. For a fixed time delay $\tau > 0$, $\lambda(\omega, \tau)$ is actually a parametric curve separates the complex plane into stable and unstable regions. Denoting $\lambda = x + \mathrm{i}y$, we get

$$\begin{cases} x = d\cos\tau\omega - \omega\sin\tau\omega, \\ y = d\sin\tau\omega + \omega\cos\tau\omega. \end{cases} \tag{S6}$$

By basic calculations, we derive that the least positive root ω_0 of the second algebraic equation $y(\omega) = 0$ in Eq. (S6) exists and satisfies $\omega_0 \in \left(\frac{\pi}{2\tau}, \frac{\pi}{\tau}\right)$. Moreover, we have $\lambda(-\omega, \tau) = \bar{\lambda}(\omega, \tau)$. The trajectory $\lambda(\omega, \tau)$, $\omega \in [-\omega_0, \omega_0]$ therefore forms a simple closed curve (denoted by Γ_τ) on the complex plane, which is symmetric to the x-axis. Figure S1 shows several parametric curve Γ_τ corresponding to different τ. Continuity stipulates that the region Ω_τ surrounded by Γ_τ is nothing but the stability region, because Eq. (S4) corresponding to the origin $\lambda = 0$ inside Ω_τ for any τ is always stable. Consequently, the equilibrium of Eq. (S1) is stable if all the eigenvalues λ_i of the community matrix $\mathbf{A}$ lie in the region Ω_τ, i.e., $\Omega_{\mathbf{A}} \subset \Omega_\tau$.

As the time delay τ increases, the area of the region Ω_τ decreases monotonically [14]. For $\tau = 0$, Ω_τ degenerates into the half plane $\{\lambda = x + \mathrm{i}y \,|\, x < d\}$. For $\tau > 0$, Ω_τ is a leaf-shaped bounded region and shrinks into a circular disk of radius d centered at the origin: $\Omega_\infty = \{\lambda = x + \mathrm{i}y \,|\, |\lambda| < d\}$ in the limit $\tau \to +\infty$. We have that $\Omega_{\mathbf{A}}$ is the estimated distribution of eigenvalues of $\mathbf{A}$ that depends mainly on the correlation coefficient ρ and the complexity α. In what follows, we provide a detailed analysis about the conditions under which the ellipse $\Omega_{\mathbf{A}}$ is a subset of the stability region Ω_τ. We remark that, in a recent work [15], the stability condition is considered heuristically by taking into account solely the relative positions of the endpoints of the ellipse to the stability region Ω_τ. Here, to be more rigorous, we will analyze the exact tangent point of $\Omega_{\mathbf{A}}$ and Ω_τ, especially for $\rho < 0$ (i.e., the predator-prey systems).

2.1.1 Cases when $\rho \geqslant 0$

For $\rho \geqslant 0$, the long axis of $\Omega_{\mathbf{A}}$ is the segment $\mathrm{Re}(\lambda) \in [-\alpha(1+\rho), \alpha(1+\rho)]$ lying on the real axis. As the complexity α increases from zero, the ellipse $\Omega_{\mathbf{A}}$ is contained inside the unit disk Ω_∞ and hence inside Ω_τ until the right endpoint of the long axis $(\alpha(1+\rho), 0)$ reaches the tangent point $(d, 0)$. It can be seen that, for $\rho \geq 0$, $(d, 0)$ is the only possible tangent point between the boundaries of $\Omega_{\mathbf{A}}$ and Ω_τ for any value of τ. As a result, when $\alpha(1+\rho) < d$, we have $\Omega_A \subset \Omega_\infty \subset \Omega_\tau$ for any time delay $\tau \geqslant 0$. On the contrary, when $\alpha(1+\rho) > d$, we get $\Omega_{\mathbf{A}} \cap \Omega_\tau^c \neq \emptyset$, which implies that there are some eigenvalue λ of $\mathbf{A}$ belonging to the unstable region for any time delay $\tau \geqslant 0$. Consequently, the absolute stability and instability conditions are respectively $\alpha < d/(1+\rho)$ and $\alpha > d/(1+\rho)$. Here the term "absolute" is used because the conditions are independent of the time delay τ. As a final result, the critical admissible complexity is found as $\alpha^* = d/(1+\rho)$ when $\rho \geqslant 0$.

2.1.2 Cases when $\rho < 0$

For $\rho < 0$, the analysis becomes more complicated because the long axis of $\Omega_{\mathbf{A}}$ lies now on the imaginary axis with the endpoints $(0, \pm\alpha(1-\rho))$. Analogously, the ellipse $\Omega_{\mathbf{A}}$ becomes larger as the complexity α increases. It is then convenient to consider three subcases.

Subcase (i): When $\alpha(1-\rho) < d$ or, equivalently, $\alpha < d/(1-\rho)$, the long axis of the ellipse $\Omega_{\mathbf{A}}$ is located inside the disk Ω_∞ centered at the origin with radius d, implying that $\Omega_{\mathbf{A}} \subset \Omega_\infty \subset \Omega_\tau$ for any time delay $\tau \geqslant 0$. In this case, the steady state is absolutely stable.

Subcase (ii): When $\alpha(1+\rho) > d$ or, equivalently, $\alpha > d/(1+\rho)$, the point $(d, 0)$ on the boundary Γ_τ of the stability region is always located inside $\Omega_{\mathbf{A}}$, resulting in absolute instability.

Subcase (iii): In addition to the subcases (i) and (ii), the complexity α lies between $d/(1-\rho)$ and $d/(1+\rho)$. Consequently, both $\Omega_A \cap \Omega_\infty^c \neq \emptyset$ and $\Omega_{\mathbf{A}} \subset \Omega_0$ hold. Remark that here we neglect the critical situations $\alpha = d/(1-\rho)$ and/or $\alpha = d/(1+\rho)$ for simplicity, but these issues will be discussed later. In this case, a fixed time delay $\tau > 0$ corresponds to a stability region Ω_τ. As the complexity α increases from $d/(1-\rho)$, we first have $\Omega_{\mathbf{A}} \subset \Omega_\tau$ yielding that the equilibrium state is stable. Then, at a critical complexity $\alpha = \alpha^*$, the two regions $\Omega_{\mathbf{A}}$ and Ω_τ intersect at a tangent point somewhere on the upper right part of the ellipse, see Fig. S1. When $\alpha > \alpha^*$, we have $\Omega_{\mathbf{A}} \cap \Omega_\tau^c \neq \emptyset$ leads to instability of the equilibrium abundances.

Also in this case, let us now consider the case when the complexity $\alpha \in (d/(1-\rho), d/(1+\rho))$ is fixed. As

Ω_τ shrinks to Ω_∞ monotonically as τ increases from zero, there is certainly a unique critical time delay $\tau_{\rm cr}$ such that the boundaries of $\Omega_{\mathbf{A}}$ and $\Omega_{\tau_{\rm cr}}$ become tangent (also at somewhere on the upper right part of the ellipse). Therefore, $\Omega_{\mathbf{A}} \subset \Omega_\tau$ as long as time delay satisfies $\tau \in [0, \tau_{\rm cr})$. Consequently, in this case, the steady state that we consider is stable for $\tau < \tau_{\rm cr}$ but becomes unstable for $\tau > \tau_{\rm cr}$, as illustrated in Fig. S1.

Figure 3**a** of the main text summarizes the analysis we carried out so far, which shows that the parameter space $\{(\alpha,\rho)\,|\,\alpha>0 \text{ and } -1\le\rho\le 1\}$ can be divided into three regions corresponding to different stability criteria as follows

$$\begin{aligned} \mathrm{I} &:= \left\{(\alpha,\rho)\,\middle|\, -1\le\rho\le 1 \text{ and } 0\le\alpha\le\frac{d}{1+|\rho|}\right\},\\ \mathrm{II} &:= \left\{(\alpha,\rho)\,\middle|\, -1\le\rho\le 1 \text{ and } \alpha\ge\frac{d}{1+\rho}\right\},\\ \mathrm{III} &:= \left\{(\alpha,\rho)\,\middle|\, -1\le\rho< 0 \text{ and } \frac{d}{1-\rho}<\alpha<\frac{d}{1+\rho}\right\}. \end{aligned} \tag{S7}$$

For any time delay $\tau \geqslant 0$, the steady state $\boldsymbol{x}\equiv\mathbf{0}$ of Eq. (S1) is always stable and unstable in regions I and II, respectively. The transition between the two regions is determined by the criterion $\alpha = d/(1+\rho)$, at which the rightmost endpoints of both $\Omega_{\mathbf{A}}$ and Ω_τ coincide as the intersecting point. However, in Region III (corresponding to the predator-prey communities), the stability depends significantly on the value of τ in an area circumscribed by the curves $\alpha = d/(1+\rho)$ and $\alpha = d/(1-\rho)$. The latter curve describes the critical case where the top endpoints of both $\Omega_{\mathbf{A}}$ and Ω_∞ coincide, which breaks the "absolute" stability.

We have seen that the case when $\rho<0$ is more complicated because $\Omega_{\mathbf{A}}$ and Ω_τ may not intersect at the four endpoints of the ellipse but some point at the upper right part (Fig. S1). Now, we are going to compute the intersecting point and its corresponding critical admissible complexity α^* or critical time delay $\tau_{\rm cr}$.

We denote the tangent point as $\lambda_{\tan} = x + \mathrm{i}y$. From Eqs. (S3) and (S6), we have the following algebraic equation

$$F_1(\rho,\tau,\omega,\alpha) := \frac{(d\cos\tau\omega-\omega\sin\tau\omega)^2}{(1+\rho)^2} + \frac{(d\sin\tau\omega+\omega\cos\tau\omega)^2}{(1-\rho)^2} - \alpha^2 = 0. \tag{S8}$$

In addition, the tangent vectors at the point $\lambda_{\tan}$ against the two boundaries $\lambda(\alpha,\rho)$ and $\lambda(\omega,\tau)$ are parallel to each other and satisfy respectively

$$\frac{x\,\mathrm{d}x}{(1+\rho)^2} + \frac{y\,\mathrm{d}y}{(1-\rho)^2} = 0,$$

and

$$\begin{cases} \dfrac{\mathrm{d}x}{\mathrm{d}\omega} = -\tau\omega\cos\tau\omega - (1+d\tau)\sin\tau\omega,\\[2ex] \dfrac{\mathrm{d}y}{\mathrm{d}\omega} = (1+d\tau)\cos\tau\omega - \tau\omega\sin\tau\omega, \end{cases}$$

which imply the second algebraic equation

$$\begin{aligned} F_2(\rho,\tau,\omega) :=& \frac{(d\cos\tau\omega-\omega\sin\tau\omega)(\tau\omega\cos\tau\omega+(1+d\tau)\sin\tau\omega)}{(1+\rho)^2}\\ &-\frac{(d\sin\tau\omega+\omega\cos\tau\omega)((1+d\tau)\cos\tau\omega-\tau\omega\sin\tau\omega)}{(1-\rho)^2} = 0. \end{aligned} \tag{S9}$$

Because of the symmetry to the real axis, we can assume the critical frequency $\omega^* \in [0,\omega_0]$. For a fixed correlation $\rho<0$, we now have two algebraic equations Eqs. (S8) and (S9) and three unknowns α,τ and ω. Therefore, with a certain time delay $\tau>0$, we are able to compute α^* and ω^* from the two equations. The former one is indeed the critical admissible complexity of the predator-prey communities. Conversely, if the complexity of the predator-prey community is given, we can also calculate $\tau_{\rm cr}$ and ω^* accordingly. In both cases, the tangent point $\lambda_{\tan}$ can then be obtained. In practice, when the system parameters are provided, we solve the critical values numerically.

Note that region III is below the α-axis, i.e., there is a critical time delay $\tau_{\rm cr}$ only when ρ is negative. This implies that the predator-prey systems possess distinct properties compared to the fully random and the mixed

ecosystem (i.e., $\rho \geqslant 0$). Figure 3**b** of the main text shows that $\tau_{\rm cr}(\alpha, \rho)$–written as a function of α and ρ–is not continuous at the boundary between regions II and III, due to the departure of the rightmost endpoint of the ellipse $\Omega_{\mathbf{A}}$ from Ω_τ when seeking the critical condition for the tangency of their boundaries.

For a fixed $\rho < 0$, we now consider $\alpha = d/(1+\rho)$ which is the boundary value of regions II and III. The corresponding ellipse $\Omega_{\mathbf{A}}$ is then tangent to the straight line $\mathrm{Re}(\lambda) = d$, which is the critical case of stability for $\tau = 0$. As τ increases from zero, $\Omega_{\mathbf{A}}$ is still fully covered by Ω_τ until their boundaries get osculated when τ reaches the value $\tau_{\rm cr}(d(1+\rho), \rho)$, the left limit value at $(d/(1+\rho), \rho)$, which can be obtained from the condition that the curvatures of both boundaries are equal at the point $\lambda = d$. Specifically, after some algebra, we obtain the following explicit expression

$$\hat{\tau}_{\rm cr} := \tau_{\rm cr}\left(\frac{d}{1+\rho}, \rho\right) = \frac{(1-\sqrt{-\rho})^2}{2d\sqrt{-\rho}}.$$

Note that $\omega \equiv 0$ is always a trivial solution of Eq. (S9) that corresponds to $\alpha = d/(1+\rho)$ and the tangent point $(d, 0)$. For the specific value $\omega = \pi/(2\tau)$, we can show that $F_2[\rho, \tau, \pi/(2\tau)] < 0$. A nontrivial positive solution $\omega \in (0, \omega_0)$ then exists under the condition $\partial F_2/\partial\omega > 0$, leading to $\tau > \hat{\tau}_{\rm cr}$.

Two remarks about the stability on the boundaries of the parameter regions I, II and III are the following. Firstly, region III is relatively open since $\tau_{\rm cr} \in (\hat{\tau}_{\rm cr}, \infty)$. Thus, the boundary curve $\{(\alpha, \rho) \,|\, \alpha = d/(1-\rho)$ and $-1 \leq \rho < 0\}$ separating regions I and III belongs to region I, so does the boundary curve $\{(\alpha, \rho) \,|\, \alpha = d/(1+\rho)$ and $-1 < \rho < 0\}$ with region II. Secondly, the segment $\{\alpha = d/(1+\rho), 0 \leq \rho \leq 1\}$ belongs to region II, because the uniform distribution of eigenvalues in the ellipse $\Omega_{\mathbf{A}}$ is an asymptotic behavior in the limit of infinite community size. For the ecosystem with finite size S, there is a nonzero probability that at least one eigenvalue of $\mathbf{A}$ is located outside $\Omega_{\mathbf{A}}$.

2.2 Distributed time delays and time shift $\hat{\tau} \geqslant 0$

Following the notation in the main text, the linearization of an ecosystem with distributed time delay is written as

$$\dot{\boldsymbol{x}}(t) = -d\boldsymbol{x}(t) + \mathbf{A}\int_0^\infty k(\tau)\boldsymbol{x}(t-\tau)\,\mathrm{d}\tau, \tag{S10}$$

where the kernel $k(\tau)$ is a probability density function that satisfies $k(\tau) \geqslant 0$ for $s \geqslant 0$ and $\int_0^\infty k(\tau)\,\mathrm{d}\tau = 1$. For $k(\tau) = \delta(\tau - c),\ c \geqslant 0$ where $\delta(\cdot)$ is the Dirac delta function, the system degenerates into the one with a discrete time delay. As introduced in the main text, there is sometimes a time shift (i.e., inherent delay) $\hat{\tau} \geqslant 0$. In such a case, the delay kernel is characterized as

$$k(\tau) = \begin{cases} 0, & 0 \leqslant \tau < \hat{\tau}, \\ k_0(\tau - \hat{\tau}), & \tau \geqslant \hat{\tau}, \end{cases} \tag{S11}$$

where $k_0(\tau)$ is a given probability density function satisfying $k_0(\tau) \geqslant 0$ for $\tau \geqslant 0$ and $\int_0^\infty k_0(\tau) = 1$.

Similar to the case discussed in Sec. 2.1, we decompose the community matrix $\mathbf{A}$ as $\mathbf{A} = \mathbf{P}\mathbf{\Lambda}\mathbf{P}^{-1}$, so it suffices to consider the following system of scalar variable through the linear transform $\boldsymbol{x} = \mathbf{P}\boldsymbol{u}$

$$\dot{u}(t) = -du(t) + \lambda\int_0^\infty k(\tau)u(t-\tau)\,\mathrm{d}\tau, \tag{S12}$$

where λ is the eigenvalue of $\mathbf{A}$ and we again omit the subscript i but emphasize that there is a characteristic equation for each eigenvalue of $\mathbf{A}$. It is required that $u(t) = 0$ is stable for all λ to guarantee the stability of $\boldsymbol{x} \equiv \mathbf{0}$ of Eq. (S12).

To analyze the stability of $u(t) = 0$ of Eq. (S12), we again leverage the ansatz $u(t) = \mathrm{e}^{zt},\ z \in \mathbb{C}$ and derive the corresponding characteristic equation as

$$H_{k(\cdot)}(z) \coloneqq z + d - \lambda K(z) = 0, \tag{S13}$$

where

$$K(z) \coloneqq \int_0^\infty \mathrm{e}^{-z\tau}k(\tau)\,\mathrm{d}\tau = \int_{\hat{\tau}}^\infty \mathrm{e}^{-z\tau}k_0(\tau-\hat{\tau})\,\mathrm{d}\tau = \int_0^\infty \mathrm{e}^{-z(v+\hat{\tau})}k_0(v)\,\mathrm{d}v = \mathrm{e}^{-z\hat{\tau}}\int_0^\infty \mathrm{e}^{-zv}k_0(v)\,\mathrm{d}v. \tag{S14}$$

Denoting $K_0(z) \coloneqq \int_0^\infty \mathrm{e}^{-z\tau}k_0(\tau)\,\mathrm{d}\tau$, we then have

$$H_{k(\cdot)}(z) = z + d - \lambda\mathrm{e}^{-z\hat{\tau}}K_0(z) = 0. \tag{S15}$$

In this work, we assume that $K_0(z)$ is well-defined and continuously differentiable, which is a generally satisfied condition for common density functions used in the context of ecology such as the Gamma distribution.

The equilibrium state of the original system is stable if, for all λ of $\mathbf{A}$, all roots of the characteristic equation $H_{k(\cdot)}(z) = 0$ have negative real parts. Analogous to the distributed-time-delay case, we can define the stability region as

$$\Omega_{k(\cdot)} \coloneqq \left\{\lambda = x + \mathrm{i}y \,\middle|\, H_{k(\cdot)}(z) = 0 \Rightarrow \mathrm{Re}(z) < 0\right\}, \tag{S16}$$

whose boundary is denoted as $\Gamma_{k(\cdot)}$. Thus, the steady state $\boldsymbol{x} \equiv \mathbf{0}$ is stable if $\Omega_{\mathbf{A}} \subset \Omega_{k(\cdot)}$.

Now, we consider the critical case $z = \mathrm{i}\omega,\ \omega \in \mathbb{R}$. When $z = \mathrm{i}\omega$, after simple calculations, we obtain

$$\hat{K}(\omega) \coloneqq K(\mathrm{i}\omega) = K_{\mathrm{R}}(\omega) + \mathrm{i}K_{\mathrm{I}}(\omega), \tag{S17}$$

with

$$K_{\mathrm{R}}(\omega) = \int_0^\infty \cos[\omega(\tau+\hat{\tau})]k_0(\tau)\,\mathrm{d}\tau, \tag{S18}$$

and

$$K_{\mathrm{I}}(\omega) = -\int_0^\infty \sin[\omega(\tau+\hat{\tau})]k_0(\tau)\,\mathrm{d}\tau. \tag{S19}$$

Considering that $\lambda = x + \mathrm{i}y \in \mathbb{C}$ and the critical case $H_{k(\cdot)}(\mathrm{i}\omega) = 0$, we obtain

$$\begin{cases} x = \dfrac{dK_{\mathrm{R}}(\omega) + \omega K_{\mathrm{I}}(\omega)}{\left|\hat{K}(\omega)\right|^2}, \\ y = \dfrac{\omega K_{\mathrm{R}}(\omega) - dK_{\mathrm{I}}(\omega)}{\left|\hat{K}(\omega)\right|^2}. \end{cases} \tag{S20}$$

For a fixed time shift $\hat{\tau}$ and a given density function $k_0(\tau)$, Eq. (S20) is indeed the parametric form $[x(\omega), y(\omega)]$ of the boundary $\Gamma_{k(\cdot)}$. In general, the shape of $\Omega_{k(\cdot)}$ and its boundary $\Gamma_{k(\cdot)}$ is sophisticated and difficult to be analyzed universally. We therefore first consider a common probability distribution, the Gamma distribution, and then provide some general results.

2.2.1 The Gamma distribution

For the Gamma distribution, the probability density function is written as

$$k_0(\tau) = \frac{\theta^{-m}}{\Gamma(m)} \tau^{m-1} \mathrm{e}^{-\tau/\theta}, \quad \tau > 0, \tag{S21}$$

where $\theta \in \mathbb{R}^+$ and $m \in \mathbb{R}^+$. Moreover, we have

$$\langle \tau \rangle := \mathbb{E}[\tau] = \int_0^{+\infty} \tau k_0(\tau) \, \mathrm{d}\tau = m\theta. \tag{S22}$$

By simple calculations, we obtain

$$\hat{K}(\omega) = \mathrm{e}^{-\mathrm{i}\omega\hat{\tau}} (1 + \mathrm{i}\omega\theta)^{-m}. \tag{S23}$$

Then, for a given time shift $\hat{\tau} > 0$, the parametric form of the boundary $\Gamma_{k(\cdot)}$ of the stability region is obtained as

$$x(\omega) + \mathrm{i}y(\omega) = \mathrm{e}^{\mathrm{i}\omega\hat{\tau}} (d + \mathrm{i}\omega)(1 + \mathrm{i}\omega\theta)^m, \tag{S24}$$

which is actually equivalent to Eq. (S20).

In the main text (Fig. 4), we consider $m = 1$ and $m = 2$ as two examples. When $m = 1$, the Gamma distribution is actually reduced into the exponential distribution as

$$k_0(\tau) = \frac{1}{\theta} \mathrm{e}^{-\tau/\theta}, \quad \tau > 0. \tag{S25}$$

In this case, when there is no time shift (i.e., $\hat{\tau} = 0$) the stability region is found as the following simple form

$$\Omega_{k(\cdot)} = \Omega_{\exp(\theta)} = \left\{ x + \mathrm{i}y \in \mathbb{C} \,\middle|\, x \leqslant d - \frac{\theta}{(1 + d\theta)^2} \, y^2 \right\}. \tag{S26}$$

Note that θ indeed delineates the average time delay. As it increases from zero, we find that the stability region changes continuously but non-monotonically. As $\theta \to 0$ or $\theta \to +\infty$, the boundary tends to the vertical line $x = d$, which corresponds to the two limiting cases.

Because we have the expression of the stability region, it is possible to calculate explicitly the critical admissible complexity. By basic algebras, we are able to show that the disk $\Omega_\infty = \{\lambda = x + \mathrm{i}y \,|\, |\lambda| < d\}$ always lies in the stability region. Consequently, the incorporated exponentially distributed time delay does not change the critical complexity of the fully random ($\rho = 0$) and the mixed ecosystems ($\rho > 0$), for which the critical admissible complexity are $\alpha^* = d$ and $\alpha^* = d/(1 + \rho)$, respectively. As for the predator-prey communities ($\rho < 0$), we need to first compute the tangent point of the boundary of $\Omega_{\exp(\theta)}$ and $\Omega_{\mathbf{A}}$, which can be found from the following algebraic equations

$$\begin{aligned} \frac{x}{(1+\rho)^2} \, \mathrm{d}x + \frac{y}{(1-\rho)^2} \, \mathrm{d}y &= 0, \\ \mathrm{d}x + \frac{2y\theta}{(1+d\theta)^2} \, \mathrm{d}y &= 0. \end{aligned} \tag{S27}$$

We finally find the intersecting point (x^*, y^*) as

$$x^* = \left(\frac{1+\rho}{1-\rho}\right)^2 \frac{(1+d\theta)^2}{2\theta}, \quad y^* = \pm(1+d\theta)\sqrt{\frac{d-x^*}{\theta}}. \tag{S28}$$

Finally, the critical admissible complexity for the predator-prey systems is obtained as

$$\begin{aligned} \alpha^* &= \sqrt{\left(\frac{x^*}{1+\rho}\right)^2 + \left(\frac{y^*}{1-\rho}\right)^2} \\ &= \frac{1+d\theta}{(1-\rho)\sqrt{2\theta}}\sqrt{1+2d-\left(\frac{1+\rho}{1-\rho}\right)^2\frac{(1+d\theta)^2}{\theta}}. \end{aligned} \tag{S29}$$

It can be verified that the critical complexity α^* changes non-monotonically as the average time delay θ increases from zero. This is actually caused by the non-monotonicity of the stability region with respect to θ.

We are able to carry out analogous investigations when $m = 2$. Because some expressions are lengthy to be provided explicitly, here we only show the boundary of the stability region, which is written in the parametric form as

$$x(\omega) = d(1-\theta^2\omega^2) - 2\theta\omega^2, \ y(\omega) = \omega(1-\theta^2\omega^2) + 2d\theta\omega, \ \omega \in \left[-\frac{\sqrt{1+2d\theta}}{\theta}, \frac{\sqrt{1+2d\theta}}{\theta}\right]. \tag{S30}$$

Again, in this case, the distributed time delay does not alter the critical complexity for ecosystems with $\rho \geqslant 0$. Moreover, it changes non-monotonically the critical complexity α^* of the predator-prey communities, see Fig. 4 of the main text.

In general, the above computations can be carry out when the density function $k(\tau)$ is given explicitly even when the time shift $\hat{\tau} > 0$. To gain further insights of the distributed time delay and to see how time shift $\hat{\tau}$ affects the critical complexity, we next provide some general properties for the ecosystems when distributed time delay are incorporated. We also discuss two cases, $\rho \geq 0$ and $\rho < 0$, separately.

2.2.2 Some general results for the cases when $\rho \geqslant 0$

For $\rho \geqslant 0$, we have the following result:

Theorem 3. *The radius-d disk $\Omega_\infty = \{\lambda = x + \mathrm{i}y \mid |\lambda| < d\}$ is contained in the stability region $\Omega_{k(\cdot)}$ for any kernel function $k(s)$. For $\rho \geq 0$, the system is therefore either absolutely stable or absolutely unstable.*

Proof. We first show that the disk Ω_∞ is always a subset of $\Omega_{k(\cdot)}$ for an arbitrary density function $k(s)$. If not, then for some $\lambda \in \Omega_\infty$, its corresponding characteristic equation $H_{k(\cdot)}(z) = 0$ has at least one root satisfying $\mathrm{Re}(z) \geqslant 0$. We thus have

$$\mathrm{Re}(z) = \mathrm{Re}(-d + \lambda K(z)) \leq -d + |\lambda K(z)| < -d + d\int_0^\infty \left|\mathrm{e}^{-z\tau}\right| k(\tau)\,\mathrm{d}\tau \leq 0,$$

which is a contradiction. This proves the first part of the theorem.

Note that, when $\lambda = d$, $z = 0$ is a trivial zero point of $H_{k(\cdot)}(z)$ which is independent of the choice of density function $k(\tau)$. The point $\lambda = d$ is therefore located on the boundary $\Gamma_{k(\cdot)}$ for any $k(\tau)$. The derivative at this point

$$\left.\frac{\partial\,\mathrm{Re}(z)}{\partial\lambda}\right|_{z=0,\lambda=d} = -\,\mathrm{Re}\left\{\frac{\partial H_{k(\cdot)}}{\partial\lambda}\Big/\frac{\partial H_{k(\cdot)}}{\partial z}\right\}\Bigg|_{z=0,\lambda=d} = \left[1 + d\int_0^\infty \tau k(\tau)\,\mathrm{d}\tau\right]^{-1} > 0$$

implies transversality and $\lambda \notin \Omega_{k(\cdot)}$ if $\lambda \in (d, d+\varepsilon)$ holds for some positive ε. Because $\Omega_{\mathbf{A}}$ is an ellipse with its long axis $[-\alpha(1+\rho), \alpha(1+\rho)]$ lying on the real axis [i.e., $\mathrm{Im}(\lambda) = 0$], either $\Omega_{\mathbf{A}} \subset \Omega_\infty \subset \Omega_{k(\cdot)}$ holds or the point $\lambda = d$ (on the boundary of $\Omega_{k(\cdot)}$) is located inside $\Omega_{\mathbf{A}}$ for any kernel $k(\tau)$. The former and the latter case corresponds to absolute stability and absolute instability, respectively. □

In light of Theorem 3, we deduce that the distributed time delay does not affect the critical complexity α^* for the ecosystems with $\rho \geqslant 0$. Therefore, we always have $\alpha^* = d/(1+\rho)$ for $\rho \geqslant 0$.

2.2.3 Some general results for the cases when $\rho < 0$

The all-or-none property (i.e., either absolute stability or absolute instability) holds for $\rho \geq 0$ because, in this case, it is only necessary to consider the relationship between the point $\lambda = d$ and the rightmost point $(\alpha(1+\rho), 0)$ of $\Omega_{\mathbf{A}}$. For the case of $\rho < 0$, the analysis is more complicated, as the intersecting point of $\Omega_{\mathbf{A}}$ and the boundary of the stability region does not occur at the point $\lambda = d$, as seen in previous examples. In this case, it is necessary to investigate properties of the stability regions $\Omega_{k(\cdot)}$. In what follows, we focus on the role played by time shift $\hat{\tau} \geqslant 0$ to see how it affects the critical complexity of the predator-prey communities.

To begin, we introduce the following lemma (c.f., Theorem 2 in Campbell and Jessop's work [16]).

Lemma 4. *If λ is real and $\lambda > d$, then system (S12) is unstable.*

Proof. Consider the real root of the characteristic equation (S13). Note that $H(0) = d - \lambda < 0$ and

$$H(z) = z + d - \lambda \int_0^\infty \mathrm{e}^{-z\tau} k(\tau)\,\mathrm{d}\tau > z + d - \lambda \geqslant 0$$

for $z \geqslant \lambda - d$. Therefore, $H(z) = 0$ has a real positive root $z \in (0, \lambda - d)$ and thus system (S12) is unstable. □

According to Eqs. (S15)and(S20), the boundary of the stability region is found in the parametric form as

$$\lambda(\omega; \hat{\tau}) = \frac{\mathrm{e}^{\mathrm{i}\omega\hat{\tau}}(d + \mathrm{i}\omega)}{\hat{K}_0(\omega)}, \tag{S31}$$

where $\hat{K}_0(\omega) := K_0(\mathrm{i}\omega) = \int_0^\infty \mathrm{e}^{-\mathrm{i}\omega s} k_0(s)\,\mathrm{d}s$, which is in fact equivalent to the Fourier transform of the density function $k_0(\tau)$ [because $k_0(\tau) = 0$ when $\tau < 0$]. The curve described by Eq. (S15) corresponds to the critical case when the characteristic equation Eq. (S13) has a pair of conjugate pure imaginary root. Note that when $\lambda = 0$, the characteristic equation has one and only one solution $z = -d < 0$, and thus the equilibrium state that we concern is stable. Consequently, the stability region $\Omega_{k(\cdot)}$ is the set of all the points that possesses a continuous path to the origin which does not intersect with the boundary $\lambda(\omega; \hat{\tau})$.

Based on our assumption, $\hat{K}_0(\omega)$ is continuous. Let ω^* be the smallest positive zero point of $K_0(\omega)$. Note that we have $\lambda(-\omega; \hat{\tau}) = \overline{\lambda(\omega; \hat{\tau})}$ implying that the boundary is symmetric to the real axis. Moreover, we also have $\lambda(0) = d$. Consequently, to analyze the stability region, we are going to consider the parametric curve defined in the interval $\omega \in [-\omega^*, \omega^*]$. In addition, if $K_0(\omega)$ has no zero point, we denote $\omega^* = +\infty$.

We then consider the tangent point of the curve $\lambda(\omega; \hat{\tau})$ and the real axis. For this purpose, we denote the smallest positive root of the equation $\mathrm{Im}[\lambda(\omega; \hat{\tau})] = 0$ as $\omega_0(\hat{\tau})$, we have that, according to Lemma 4, if $\omega_0(\hat{\tau})$ exists and $\omega_0(\hat{\tau}) < \omega^*$, then $\lambda(\omega_0; \hat{\tau})$ must be negative, i.e., $\arg[\lambda(\omega_0; \hat{\tau})] = \pi$. In such a case, the stability region $\Omega_{k(\cdot)}$ is closed.

We have already seen that the stability region is open for the exponentially distributed delay without time shift. This means that ω_0 may not exists for arbitrary $k_0(\tau)$ when there is no time shift (i.e., $\hat{\tau} = 0$). In fact, by introducing sufficiently large time shift $\hat{\tau} > 0$, the existence of ω_0 can be guaranteed by the following lemma.

Lemma 5. *There is a $\underline{\tau} \geq 0$ such that, for any $\hat{\tau} > \underline{\tau}$, $\omega_0(\hat{\tau})$ exists and $\omega_0(\hat{\tau}) < \omega^*$. Specifically, if $\omega_0(0) < \omega^*$ exists, then $\underline{\tau} = 0$.*

Proof. First, we have $\lambda(\omega; \hat{\tau}) = \mathrm{e}^{\mathrm{i}\omega\hat{\tau}}\lambda(\omega; 0)$ for all ω and $\hat{\tau}$. This implies that

$$\arg[\lambda(\omega; \hat{\tau})] = \omega\hat{\tau} + \arg[\lambda(\omega; 0)].$$

If $\omega_0(0) < \omega^*$ exists, then for any $\hat{\tau} > 0$, we have

$$\arg[\lambda(\omega_0(0); \hat{\tau})] = \omega_0(0)\hat{\tau} + \arg[\lambda(\omega_0(0); 0)] = \omega_0(0)\hat{\tau} + \pi > \pi.$$

We also have $\arg[\lambda(0; \hat{\tau})] = \arg(d) = 0$ for any $\hat{\tau}$. Thus, by continuity of $\lambda(\omega; \hat{\tau})$, we have that, for any $\hat{\tau}$, $\omega_0(\hat{\tau})$ exists and $\omega_0(\hat{\tau}) < \omega_0(0) < \omega^*$. Consequently, $\underline{\tau} = 0$.

Now suppose $\mathrm{Im}[\lambda(\omega,0)] \neq 0$ for $\omega \in (0,\omega^*)$. For any $\hat{\tau} \geqslant 0$, from Eq. (S31), after some algebra, we have

$$\left.\frac{\mathrm{d}\lambda}{\mathrm{d}\omega}\right|_{\omega=0} = \mathrm{i}(1+d\hat{\tau}).$$

We thus have that $\lambda(\omega;0)$ lies in the upper half plane for $\omega \in (0,\omega^*)$ according to continuity, i.e., $\arg[\lambda(\omega;0)] \in (0,\pi)$. Choosing $0 < \tilde{\omega} < \omega^*$, we have

$$\arg[\lambda(\tilde{\omega};\hat{\tau})] = \tilde{\omega}\hat{\tau} + \arg[\lambda(\tilde{\omega};0)] > \tilde{\omega}\hat{\tau}. \tag{S32}$$

Now we take $\underline{\tau} = \pi/\tilde{\omega}$. Then, for $\hat{\tau} > \underline{\tau}$ and we have $\arg[\lambda(\tilde{\omega};\hat{\tau})] > \pi$, implying that there exists a $\omega_0 < \tilde{\omega} < \omega^*$ such that $\arg[\lambda(\omega_0,\hat{\tau})] = \pi$. Here, we use again the continuity of $\lambda(\omega;\hat{\tau})$. This completes the proof. □

For simplicity, hereafter we let $\underline{\tau}$ be the infimum of all those $\underline{\tau}$ satisfying Lemma 5. Having proved that $\omega_0(\tau)$ is well-defined, we are now going to discuss the monotonicity and convergence of the stability region with respect to the increasing of time shift $\hat{\tau}$. As we will see, the monotonicity is different from the case when the average time delay $\langle\tau\rangle$ increases. We first have the following lemma.

Lemma 6. *As $\hat{\tau}$ increases in the interval $(\underline{\tau},\infty)$, $\omega_0(\hat{\tau})$ decreases monotonically and converges to zero. Moreover, the endpoint $\lambda(\omega_0(\hat{\tau});\hat{\tau})$ of the boundary $\Gamma_{k(\cdot)}$ tends to $\lambda = -d$.*

Proof. For any $\delta > 0$, the relation $\lambda(\omega;\hat{\tau}+\delta) = \mathrm{e}^{\mathrm{i}\delta\omega}\lambda(\omega;\hat{\tau})$ implies that

$$\arg[\lambda(\omega_0(\hat{\tau});\hat{\tau}+\delta)] = \omega_0(\hat{\tau})\delta + \arg[\lambda(\omega_0(\hat{\tau});\hat{\tau})] = \omega_0(\hat{\tau})\delta + \pi > \pi.$$

Thus, there exists a $\omega_0(\hat{\tau}+\delta)$ that is less than $\omega_0(\hat{\tau})$ such that $\arg[\lambda(\omega_0(\hat{\tau}+\delta);\hat{\tau}+\delta)] = \pi$. Consequently, we deduce that $\omega_0(\hat{\tau})$ is a monotonically decreasing function.

For any $\epsilon > 0$, we take $\hat{\tau} = \pi/\epsilon$. Then,

$$\arg[\lambda(\epsilon;\hat{\tau})] > \epsilon\hat{\tau} + \arg[\lambda(\epsilon;0)] > \pi.$$

Therefore, $\omega_0(\hat{\tau})$ exists and satisfies $\omega_0(\hat{\tau}) < \epsilon$, which implies that $\lim_{\hat{\tau}\to\infty}\omega_0(\hat{\tau}) = 0$. Because $|\lambda(\omega;\hat{\tau})|^2 = (d^2+\omega^2)/|K_0(\omega)|^2$, it follows that $\lim_{\hat{\tau}\to\infty}|\lambda(\omega_0(\hat{\tau});\hat{\tau})| = d$. Also, $\lambda(\omega_0(\hat{\tau});\hat{\tau})$ must be negative according to Lemma 4. Therefore, it converges to $-d$ as . This completes the proof. □

From Lemma 6, we define $\omega_0(\underline{\tau}) = \lim_{\hat{\tau}\to\underline{\tau}^+}\omega_0(\hat{\tau})$. We then have $\omega_0(\hat{\tau}) < \omega_0(\underline{\tau}) \leq \omega^*$ for any $\hat{\tau} > \underline{\tau}$. We further define the following real function

$$F(\omega) := |\lambda(\omega;\hat{\tau})|^2 = \frac{\omega^2+d^2}{|K_0(\omega)|^2}.$$

The next result of our analysis is the following theorem about $\Omega_{\hat{\tau}}$ that describes the stability region for different time shift $\hat{\tau}$.

Theorem 7. *There exists $\eta \geqslant 0$ such that for $\hat{\tau} > \eta$, $\Omega_{\hat{\tau}}$ shrinks monotonically as $\hat{\tau}\to\infty$ and the limiting set is the disk $\Omega_\infty = \{\lambda \in \mathbb{C} \mid |\lambda| \leqslant d\}$. Further, if $F(\omega)$ is strictly monotonic in the interval $(0,\omega_0(\underline{\tau}))$, then $\eta = \underline{\tau}$ and $\Omega_{\hat{\tau}}$ is globally monotonic for $\hat{\tau} \in (\underline{\tau},\infty)$.*

Proof. The continuous differentiability of $K_0(\omega)$ stipulates that $F(\omega)$ is strictly monotonically increasing in some interval $(0,\epsilon)$ close to $\omega = 0$, because its numerator is monotonically increasing while the denominator is the energy spectrum of $k_0(s)$ for which $\omega = 0$ yields a maximal value. Lemma 6 implies that there exists a $\eta > 0$ such that for $\hat{\tau} > \eta$, $\omega_0(\hat{\tau}) < \epsilon$ holds and thus $F(\omega)$ is monotonically increasing in the interval $(0,\omega_0(\eta))$.

When there are two pairs $(\hat{\tau}_i,\omega_i)$, $i = 1,2$ satisfying $\hat{\tau}_i > \eta$, $0 < \omega_i < \omega_0(\hat{\tau}_i)$ such that $\lambda(\omega_1;\hat{\tau}_1) = \lambda(\omega_2;\hat{\tau}_2)$, i.e.,

$$\frac{\mathrm{e}^{\mathrm{i}\omega_1\hat{\tau}_1}(d+\mathrm{i}\omega_1)}{K_0(\omega_1)} = \frac{\mathrm{e}^{\mathrm{i}\omega_2\hat{\tau}_2}(d+\mathrm{i}\omega_2)}{K_0(\omega_2)},$$

taking absolute values of both sides implies that $F(\omega_1) = F(\omega_2)$. Because $F(\omega)$ is a monotonically increasing function in the interval $(0, \omega_0(\eta))$, we must have $\omega_1 = \omega_2$, and thus have $\tau_1 = \tau_2$. This means that, for different $\hat{\tau}$ in $\tau \in (\eta, \infty)$, the boundary of the stability region $\Omega_{\hat{\tau}}$ never intersect each other except at the trivial point $\lambda = d$.

Note that the endpoint $\lambda(\omega_0(\hat{\tau}); \hat{\tau}) = -\sqrt{F(\omega_0(\hat{\tau}))}$ is monotonically increasing with respect to $\hat{\tau}$. Because $\eta < \hat{\tau}_1 < \hat{\tau}_2$ implies $\lambda(\omega_0(\hat{\tau}_1); \hat{\tau}_1) < \lambda(\omega_0(\hat{\tau}_2); \hat{\tau}_2)$ and the boundary of $\Omega_{\hat{\tau}_1}$ and $\Omega_{\hat{\tau}_2}$ never intersect, we conclude that $\Omega_{\hat{\tau}_2} \subset \Omega_{\hat{\tau}_1}$.

We now consider the asymptotic region of $\Omega_{\hat{\tau}}$. From Theorem 3 and Lemma 6, we have $\lambda(\omega_0(\hat{\tau}); \hat{\tau}) < -d$ and $\lim_{\hat{\tau} \to +\infty} \lambda(\omega_0(\hat{\tau}); \hat{\tau}) = -d$. Moreover, $\lambda = -d$ belongs to the boundary of the limiting set Ω_∞ which always lies inside $\Omega_{\hat{\tau}}$. Therefore, for any $\hat{\tau} > \eta$, the point $\lambda(\omega; \hat{\tau})$ on the boundary of the stability region $\Omega_{\hat{\tau}}$ satisfies $|\lambda(\omega; \hat{\tau})| = \sqrt{F(\omega)}$, which is bounded between d and $|\lambda(\omega_0(\hat{\tau}))|$, i.e.,

$$\{\lambda \in \mathbb{C} \mid |\lambda| \leqslant d\} \subset \Omega_{\hat{\tau}} \subset \{\lambda \in \mathbb{C} \mid |\lambda| \leqslant |\lambda(\omega_0(\hat{\tau}))|\}.$$

It then follows that $\lim_{\hat{\tau} \to +\infty} \Omega_{\hat{\tau}} = \Omega_\infty = \{\lambda \in \mathbb{C} \mid |\lambda| \leqslant d\}$.

Finally, if $F(\omega)$ is monotonic in the interval $(0, \omega_0(\underline{\tau}))$, then it can be checked that the analysis above holds for $\hat{\tau} > \underline{\tau}$ and then $\eta = \underline{\tau}$. This completes the proof. □

With Theorem 7, we are able to further analyze the stability of the ecosystems with the correlation parameter $\rho < 0$. Recall that, in this case, the distribution of the eigenvalues of the community matrix $\mathbf{A}$ is a vertically stretched ellipse, whose long axis $[-\mathrm{i}\alpha(1-\rho), \mathrm{i}\alpha(1-\rho)]$ lies on the imaginary axis. As the complexity α of the predator-prey communities increases from zero, we have $\Omega_{\mathbf{A}} \subset \Omega_\infty \subset \Omega_{k(\cdot)}$ when $\alpha < d/(1-\rho)$. As a result, the equilibrium state that we concern is absolutely stable, i.e., the conclusion is independent of the distributed delay. As α increases further, we have that $\Omega_{\mathbf{A}} \cap \Omega_\infty^c \neq \emptyset$. Moreover, according to Theorem 7, when $\hat{\tau} > \eta$, the stability region shrinks monotonically to Ω_∞. Therefore, there is a critical value $\hat{\tau}_{\text{cr}}$ such that $\Omega_{\mathbf{A}} \subset \Omega^c$ (implying stable equilibrium state) when $\hat{\tau} < \hat{\tau}_{\text{cr}}$ and $\Omega_{\mathbf{A}} \cap \Omega_{k(\cdot)}^c \neq \emptyset$ (indicating unstable equilibrium state) when $\hat{\tau} > \hat{\tau}_{\text{cr}}$. Consequently, as the complexity α increases and exceeds $d/(1-\rho)$, the ecosystem enters an uncertain region where the stability of the steady state depends on the value of time shift $\hat{\tau}$. When α increases further and exceeds $d/(1+\rho)$, we have that $\Omega_{\mathbf{A}} \cap \Omega_{k(\cdot)}^c \neq \emptyset$ for all $\hat{\tau}$ because the short (horizontal) axis of the ellipse $\Omega_{\mathbf{A}}$ crosses the rightmost point of $\Omega_{k(\cdot)}$, i.e., $\lambda = d$. As a consequence, the equilibrium state we concern is absolutely unstable. We now summarize the analysis into the following theorem.

Theorem 8. *When $\rho < 0$, as the complexity α increases, the state of the concerned equilibrium state changes from being absolutely stable through an uncertain region to being absolutely unstable. Moreover, in the uncertain region, there is a critical time shift $\hat{\tau}_{cr}$ separates the equilibrium state into stable and unstable state.*

We remark that the critical threshold of α that separates the uncertain region and the absolutely-unstable region may not always be $d/(1+\rho)$. It depends on the choice and the properties of specific density function $k_0(\tau)$. For instance, when $\underline{\tau} = \eta = 0$ for a given $k_0(\tau)$, the stability region shrinks monotonically to Ω_∞ in a global manner. That is, the greatest stability region corresponds to the case when time shift $\hat{\tau} = 0$. In this case, if we further have that $\Omega_{\mathbf{A}} \cap \Omega_{k(\cdot)}^c \neq \emptyset$ when $\alpha = \tilde{\alpha} < d/(1+\rho)$ and $\hat{\tau} = 0$, then, the concerned steady state is absolutely unstable whenever $\alpha > \tilde{\alpha}$.

Based on Theorems 3and 8, we now conclude that the role played by time shift $\hat{\tau}$ is analogous to that by the discrete time delay τ. Specifically, when $\rho \geqslant 0$, the discrete or distributed time delays do not alter the critical complexity of the ecosystems. As for $\rho < 0$, there is an uncertain region where the amount of time delay plays significant role in determining the critical admissible complexity. In practice, for specific discrete or distributed time delays, the critical value can be calculated.

3 Detailed settings for numerical simulations

In previous sections, we provide some analytical results for the considered ecosystems with either discrete or distributed time delays. To verify our theoretical results, we need also to carry out numerical simulations. In fact, most of the results for comparison and verification are provided in the main text. Here, we give below detailed settings for numerical investigations.

3.1 Configure the three representative types of ecosystems

We refer to the previous work [17] to construct the community matrix $\mathbf{A}$ for the three representative types of ecosystems. Following the notation mentioned in the main text, we set $C \in (0,1]$ as the parameter characterizing the sparsity of the community. Denoting by ϕ, ϕ_+ and ϕ_- the probability density function of a Gaussian distribution $\mathcal{N}(0,\sigma^2)$ and the corresponding distributions with positive and negative absolute values $\pm\left|\mathcal{N}(0,\sigma^2)\right|$, respectively. In a fully random community, each element a_{ij} is drawn independently from $\mathcal{N}(0,\sigma^2)$ with probability C and is zero with probability $1-C$, i.e., the probability density function of a_{ij} is

$$p_{\mathrm{random}}(a_{ij}) = (1-C)\delta(a_{ij}) + C\phi(a_{ij}), \tag{S33}$$

where $\delta(\cdot)$ is the Dirac delta function.

For the mixed ecosystems, each pair of interactions (a_{ij}, a_{ji}) is drawn independently from $\left(\left|\mathcal{N}(0,\sigma^2)\right|, \left|\mathcal{N}(0,\sigma^2)\right|\right)$ with probability $C/2$, and from $\left(-\left|N(0,\sigma_0^2)\right|, -\left|N(0,\sigma_0^2)\right|\right)$ with the same probability $C/2$, and is $(0,0)$ with probability $1-C$. Thus, the probability density function is

$$p_{\mathrm{mixture}}(a_{ij}, a_{ji}) = (1-C)\delta(a_{ij})\delta(a_{ji}) + \frac{C}{2}\phi_+(a_{ij})\phi_+(a_{ji}) + \frac{C}{2}\phi_-(a_{ij})\phi_-(a_{ji}). \tag{S34}$$

For the predator-prey communities, each pair of interactions (a_{ij}, a_{ji}) is drawn independently with equal probability $C/2$ from $\left(\left|\mathcal{N}(0,\sigma^2)\right|, -\left|\mathcal{N}(0,\sigma^2)\right|\right)$ and $\left(-\left|\mathcal{N}(0,\sigma^2)\right|, \left|\mathcal{N}(0,\sigma^2)\right|\right)$, and is $(0,0)$ with probability $1-C$. Thus, the density function is

$$p_{\mathrm{predator-prey}}(a_{ij}, a_{ji}) = (1-C)\delta(a_{ij})\delta(a_{ji}) + \frac{C}{2}\phi_+(a_{ij})\phi_-(a_{ji}) + \frac{C}{2}\phi_-(a_{ij})\phi_+(a_{ji}). \tag{S35}$$

For simplicity, we assume that the self-interaction instantaneous, and they are considered to be the same as d in this work. Therefore, we set $a_{ii} = 0$ for all the community matrices. The above configurations yield that the interactions a_{ij} and a_{ji} of the mixed ecosystems are either both positive (indicating mutualism) or both negative (indicating competition), while those a_{ij} and a_{ji} of the predator-prey communities always have opposite signs if they are non-zero. The construction procedure also guarantees that the sparsity of the community matrix $\mathbf{A}$ (i.e., number of non-zero elements) is C and satisfies the conditions in Eq. (S2). Consequently, the complexity of each constructed ecosystem is $\alpha = \sqrt{SC}\sigma$.

Once the community matrix $\mathbf{A}$ has been configured, we are able to calculate the corresponding correlation coefficient ρ. We get $\rho_0 = 0$, $\rho_+ = 2/\pi$, and $\rho_- = -2/\pi$ for the ecosystems with fully random, mixed, and predator-prey type of communities, respectively. Note that the correlation coefficients ρ is constant and independent of other parameters. The specific values of these coefficients mean that the three types of systems represent different kinds of ensemble mutual patterns between any two species in real ecological networks, corresponding to the balanced, positive, and negative relationships, respectively.

3.2 Estimating admissible capacity $S_{\max}$

When other parameters are fixed, the admissible capacity of a network is denoted by $S_{\max}$ and defined as the largest value S make the concerned equilibrium state stable. According to the theoretical result, such a value classifies the equilibrium states into stable and unstable ones in a "binary" manner. However, this is reliable only when the network size is sufficiently large. In practice, we also want to find $S_{\max}$ for the community size

is relatively small. In such cases, due to the the stochastic nature of the community matrix $\mathbf{A}$, the equilibrium state of a specific type of ecosystem is either stable or unstable for a fixed value of S can only be determined in a probabilistic sense. We thus apply a statistical method to estimate numerically the critical value $S_{\max}$.

For a given set of parameter values, we first obtain the theoretical prediction of $S_{\max}$. For each S value around this prediction, we perform numerical simulations for sufficient number of times (e.g., 100) when generating the community matrix $\mathbf{A}$. For each $\mathbf{A}$ we determine the stability of the equilibrium state and use these results to estimate the probability of stability, p_S. Finally, we obtain an estimation of the critical value of $S_{\max}$ by fitting with a probit regression model

$$\Phi^{-1}(p_S) = \beta(S - S_{\max}),$$

where Φ^{-1} is the inverse of the standard Gaussian cumulative distribution function.

3.3 Corresponding results for discrete time delays

We first consider the case where the interactions possess a discrete time delay τ. From our analytical result (S7) about the regions of stability, we see that the fully random and mixed communities ($\rho \geqslant 0$) are either absolutely stable or absolutely unstable, depending on the value of $\alpha = \sqrt{SC}\sigma$. The transition (between the stable and unstable regions) occurs at $\alpha = d$ and $\alpha = \pi d/(\pi+2)$ for the fully random and mixed communities, respectively (see also Fig. 3 of the main text). For instance, in simulations, for $C = 0.2$, $\sigma_0 = 0.5$ and $d = 3$, the maximal admissible capacity are approximately $S_{\max} = 180$ and $S_{\max} = 67$ for the fully and mixed communities, respectively.

While discrete time delay does not affect the admissible complexity α (or the maximal capacity $S_{\max}$) for the fully random and mixed communities, it does play a significant role for the predator-prey ones. In particular, for the predator-prey communities, because $\rho_- = -2/\pi < 0$ region III emerges for complexity $\alpha \in (\pi d/(\pi + 2), \pi d/(\pi - 2))$. When the complexity α of the predator-prey communities is assigned in this interval, time delay τ contributes to determining the stability. Moreover, there is a critical value of time delay τ_{cr}, which relates to the complexity α, and can be obtained numerically as discussed in previous sections. Conversely, the admissible complexity or the network capacity also depend on the value of time delay in region III. This is the essential difference between the predator-prey communities and those with fully random or mixed interactions.

In general, an increment in the time delay plays a negative role for the admissible complexity or the network capacity of the predator-prey systems. Recall that the admissible complexity for the fully random ecosystem is $\alpha = d$. Then, given $\alpha = d$ we can find $\tau_{\text{cr}}(\alpha, \rho) = \tau_{\text{cr}}(d, \rho)$ where the predator-prey communities and the fully random ones possess the same admissible complexity. For $\tau > \tau_{\text{cr}}(d, \rho)$, the critical admissible complexity of the predator-prey communities become less than d. As a result, if other parameters are fixed, the maximal network capacity that a stable predator-prey community accommodate will be smaller than that of a fully random one. For different types of ecosystems, we carry out simulations for different values of the time delay τ and find that the results agree with those from theoretical analysis, see Fig. 5 of the main text. More results are provided in Table S1. For instance, for $\tau = 0.5$, numerical computation gives $S_{\max} = 96$ for the predator-prey communities, which is much smaller than $S_{\max} = 180$ for the fully randomly ecosystems, yet still larger than $S_{\max} = 67$ for the mixed ones. More importantly, comparing with ecosystems without any time delay, the hierarchy of stability for the three types of systems changes for $\tau > 0.165$ - see Table S1 for more details, where the parameters are $C = 0.2$, $\sigma_0 = 0.5$, and $d = 3$.

4 Effects of other realistic factors

4.1 Robustness of altered complexity hierarchy with respect to heterogeneous time delays

We carry out a robustness analysis with respect to variations in the time delay. Considering perturbations to the time delays yields the following linearized differential equation

$$\dot{x}_i(t) = -dx_i(t) + \sum_{j=1}^{S} a_{ij} x_j(t - \tau_{ij}), \quad i = 1, \ldots, S.$$

In simulations, each τ_{ij} is drawn independently from a uniform distribution $\mathcal{U}(\tau - \delta, \tau + \delta)$. For a reasonable comparison of the effects of perturbations for the heterogeneous time delays, we take cv_τ, the coefficient of variation of the perturbation distribution, as a control parameter such that $\delta = \sqrt{3}\tau\mathrm{cv}_\tau$. Rather than calculating the distribution of the eigenvalues of the community matrix $\mathbf{A}$ and comparing their position with the stability region Ω_τ , we estimate the probability of stability directly from the simulated trajectories. The reason is that it is difficult to establish the corresponding stability theory from the distribution of the community eigenvalues with heterogeneous time delays. In simulations, given the values of S, τ and cv_τ, we generate the community matrix $\mathbf{A}$ and a set of time delays $\{\tau_{ij}\}$ for a sufficiently large number of times (e.g., 100 times) and compute the Lyapunov exponents from the simulated trajectories. The probability of stability is then estimated from the frequency of the negative Lyapunov exponents. Note that cv_τ can never be greater than $\sqrt{3}/3$ in order to guarantee non-negative time delays.

The numerical results indicate strong robustness against heterogeneous time delays, as shown in Fig. S2. Even for large perturbations with $\mathrm{cv}_\tau = 1/2$, which means that the values of τ_{ij} are taken to be very different from the value τ (e.g., from the interval $[0.13\tau, 1.87\tau]$), the trends of variation in calculated capacity $S_{\max}$ do not change appreciably. We find that the capacity is especially robust for the fully random and mixed ecosystems which is consistent with the analytical result that those values do not depend on the values of the time delay.

4.2 Effects of different probability distributions for interaction strength on the stability hierarchy

In our analysis and computations so far, the elements a_{ij} of the community matrix $\mathbf{A}$ are drawn from the Gaussian distribution $\mathcal{N}(0, \sigma^2)$. Here we consider the situation where the distribution is no longer fixed as Gaussian but can be other symmetric probability density function with zero mean and variance σ^2. For a fixed value of the variance σ^2, different probability density functions indeed lead to different interacting strength $\mathbb{E}[|a_{ij}|]$. To study the effects of the interacting strength on the hierarchical complexity, we focus mainly on the predator-prey communities because the fully random or mixed ecosystems are either absolutely stable or absolutely unstable.

From the configuration of the predator-prey communities (S35), we have $(\mathbb{E}[|a_{ij}|])^2 = |\rho|\,\sigma^2$ due to the symmetry of the probability density function of a_{ij}, which can be verified numerically. As a result, for a fixed value of σ, increasing or decreasing the interacting strength $\mathbb{E}[|a_{ij}|]$ is equivalent to changing the absolute value of the correlation $|\rho|$. The role of varying the interacting strength in our analysis is thus assessed by the variation in the correlation coefficient ρ.

For comparison with the Gaussian ensemble, we now consider the ensemble of a uniform distribution $\mathcal{U}[-\sqrt{3}\sigma, \sqrt{3}\sigma]$ with zero mean and standard derivation σ, for which the correlation coefficients between a_{ij} and a_{ji} are $\rho_0 = 0$, $\rho_+ = 0.75$, and $\rho_- = -0.75$ for the fully random, mixed, and predator-prey communities, respectively. To compare the two ensembles, we first denote $\rho_+^u = 0.75$ and $\rho_+^n = 2/\pi$ for the uniform and Gaussian distribution, respectively, for positive ρ. Thus, we have $\rho_+^u > \rho_+^n$. The correlation of the pair of interactions in structured ecosystems are stronger for the uniform distribution than that for the Gaussian distribution. As already revealed by our theoretical analysis, the stability criteria for the fully random or mixed

ecosystems are independent of the time delay. For the former one, the critical admissible complexity is always found as $\alpha^* = d$ which is invariant for different ensembles. As for the mixed ecosystems, the critical complexity becomes $\alpha^* = d/(1+\rho)$. Specifically, we have $\alpha^* = 4d/7$ for the uniform distributed a_{ij}, which is smaller than $\alpha^* = \pi d/(\pi+2)$ for the case of Gaussian ensemble. Consequently, for the mixed ecosystems, the Gaussian distributed interactions accommodate greater complexity.

For predator-prey communities, the stability criterion depends highly on the time delay. To gain insights, we consider here two extreme cases for which the value of τ is near or far from zero. Firstly, when τ is sufficiently small (near zero), the critical complexity of a given predator-prey community is approximately equivalent to that for the case when there is no time delay, i.e., $\tau = 0$. In such a case, we have $\alpha^* \approx d/(1+\rho) = 4d$ for the uniformly distributed interactions and $\alpha^* \approx \pi d/(\pi - 2)$ for the Gaussian ensemble. Because of the inequality $4d > \pi d/(\pi - 2)$, the predator-prey communities with uniformly distributed interactions have greater admissible complexity than those with the Gaussian distribution. In the opposite extreme case where τ is sufficiently large, the phase transition occurs near the boundary between regions I and III, where the critical complexity becomes $\alpha^* = 4d/7$ and $\alpha^* = \pi d/(\pi+2)$ for the uniform and the Gaussian distributions, respectively. Consequently, the order of critical admissible complexity for the predator-prey communities with two different ensembles of interactions is analogous to that for the mixed ecosystems.

The insights so gained indicate that there is a critical value of the time delay where the two types of predator-prey ecosystems, one with the uniform and the other with the Gaussian distribution of interactions, accommodate the same critical complexity. In particular, in region III with a fixed complexity α, the stability of the equilibrium state is determined by whether the inequality $\tau < \tau_{\rm cr}$ holds. As stated in previous sections, we are able to calculate the value of $\tau_{\rm cr}$ once the complexity α and the correlation coefficient ρ is known. Conversely, if time delay τ and correlation ρ is known, we are also able to compute the critical complexity α^*. Consequently, for a specific ρ, the critical complexity α^* is written as a function of time delay τ, i.e., $\alpha^*(\tau;\rho)$. The analysis now boils down to determining the sign and zero points of the function

$$\Delta\alpha(\tau) = \alpha^*\left(\tau; -\frac{2}{\pi}\right) - \alpha^*\left(\tau; -\frac{3}{4}\right).$$

Numerical searching indicates that there is a unique zero point, which is supported by direct simulations of the dynamics, as shown in Fig. S3a. For the case of distributed time delay following, e.g., the Gamma distribution with $\theta = 0.05$ and $m = 2$, the function $\Delta\alpha$ is always positive, implying that the predator-prey communities with a greater value of $|\rho|$ have a less critical complexity α^* or system's capacity $S_{\max}$, as shown in Fig. S3b. This is due to the fact that the time delay with the kernel $k(s)$ is still considerable even when there is no time shift, i.e., $\hat{\tau} = 0$.

To summarize, increasing the interacting strength $\mathbb{E}[|a_{ij}|]$ or, equivalently, the value of $|\rho|$ diminish the complexity or capacity of the mixed ecosystems and enhances those of the predator-prey type ecosystems with a small time delay. However, when time delay becomes greater, sufficiently large interacting strength mitigates the complexity or capacity of the predator-prey communities.

5 Extended ecosystems with more interacting structures

We validate our analysis in the presence of time delay using a number of realistic ecological systems with competitive, mutualistic, or predator-prey type of interactions.

5.1 Competitive and mutualistic ecosystems

For mutualistic or competitive ecosystems, each off-diagonal elements a_{ij} of the community matrix $\mathbf{A}$ is drawn from a non-negative or a non-positive distribution, respectively, while the diagonal elements are set to zero as previously discussed. The configuration of the competitive and mutualistic coupling matrices are analogous to that for systems with mixed interactions, except that each pair of (a_{ij}, a_{ji}) is drawn with probability C only from $\left(\left|\mathcal{N}(0,\sigma^2)\right|, \left|\mathcal{N}(0,\sigma^2)\right|\right)$ for the mutualistic systems and from $\left(-\left|\mathcal{N}(0,\sigma^2)\right|, -\left|\mathcal{N}(0,\sigma^2)\right|\right)$ for the competitive systems. We thus have the average interacting strength is $\mu = \pm\sqrt{2/\pi}C\sigma$ ("+" for mutualistic and "−" for competitive systems), the variance becomes $\tilde{\sigma}^2 = (1-2C/\pi)C\sigma^2$, and the correlation coefficient is $\rho = (1-C)/(\pi/2-C)$.

To analyze the eigenvalues of the community matrix $\mathbf{A}$ for mutualistic and competitive systems, it is sufficient to study the mutualistic one because for the competitive one we only need to multiply the matrix by -1. Therefore, for the following analysis, we have $a_{ij} \geqslant 0$ for all i and j. We again decompose $\mathbf{A}$ as $\mathbf{A} = \mathbf{P\Lambda P}^{-1}$. The columns $\boldsymbol{v}_i$ of $\mathbf{P}$ are the right eigenvectors and the rows $\boldsymbol{w}_i^\top$ of $\mathbf{P}^{-1}$ are the left eigenvectors of $\mathbf{A}$ with the inner product $\boldsymbol{w}_i^\top \boldsymbol{v}_i = \mathbf{1}$ where $\mathbf{1} = [1, \cdots, 1]^\top$. For a system with sufficiently large community size S, the sums of each row and each column have approximately the same value $(S-1)\mu$. Associated with the eigenvalue $\lambda_1 = (S-1)\mu$, both the right and left eigenvectors are $\boldsymbol{v}_1 = \boldsymbol{w}_1 = \mathbf{1}/\sqrt{S}$. To compute the distribution of the remaining eigenvalues, we note that the decomposition

$$\mathbf{A} = \mathbf{P\Lambda P}^{-1} = (S-1)\mu \cdot \frac{\mathbf{1}}{\sqrt{S}}\frac{\mathbf{1}^\top}{\sqrt{S}} + \sum_{i\geq 2}^{S} \lambda_i \boldsymbol{v}_i \boldsymbol{w}_i^\top$$

implies

$$\mathbf{A} - \mu\mathbf{1}\mathbf{1}^\top = -\mu \cdot \frac{\mathbf{1}}{\sqrt{S}}\frac{\mathbf{1}^\top}{\sqrt{S}} + \sum_{i\geq 2}^{S} \lambda_i \boldsymbol{v}_i \boldsymbol{w}_i^\top,$$

which is the spectrum decomposition of $\mathbf{A} - \mu\mathbf{1}\mathbf{1}^\top$. As a result, $\lambda_2, \cdots, \lambda_S$ are also eigenvalues of the matrix $\mathbf{A} - \mu\mathbf{1}\mathbf{1}^T$, which has the diagonal element $-\mu$ and zero mean off-diagonal elements. According to the elliptic law, except $\lambda_1 = -\mu$, the remaining eigenvalues are distributed uniformly in the ellipse

$$\Omega = \left\{ \lambda = x + \mathrm{i}y \in \mathbb{C} \,\middle|\, \frac{x^2}{(1+\rho)^2} + \frac{y^2}{(1-\rho)^2} \leq \alpha^2 \right\}$$

asymptotically, where $\alpha = \sqrt{S}\tilde{\sigma}$. Hence, for matrix $\mathbf{A}$, except for $\lambda_1 = (S-1)\mu$, the remaining eigenvalues follow asymptotically a uniform distribution on the ellipse Ω. For large community size S, λ_1 is the leading eigenvalue since $\lambda_1 \sim \mathcal{O}(S)$ and $\lambda_i \sim \mathcal{O}(\sqrt{S})$ for $i \geqslant 2$. Analogously, the competitively community matrix has the leading eigenvalue $\lambda_1 = -(S-1)\mu$ and its remaining eigenvalues are distributed uniformly on Ω.

We can now analyze the critical admissible complexity of the two ecosystems. For the mutualistic systems, to guarantee the stability of the concerned equilibrium state, we need to have $\lambda_1 < d$ because the distribution of other eigenvalues Ω is always located inside the circle Ω_∞ (with radius d and centered at the origin). Specifically, we obtain that the maximal capacity is (approximately) $S_{\max} = 1 + \sqrt{\pi/2} \cdot [d/(C\sigma)]$, which is independent of the amount of time delay. However, for the competitive ecosystems, a greater time delay does affect its maximal capacity. To illustrate this, we consider the systems with a discrete time delay τ, for which the stability criteria that both the leading eigenvalue $\lambda_1 = -(S-1)\mu$ and the ellipse Ω are located inside Ω_τ result in

$$\begin{cases} |\lambda_1| = (S-1)\mu < |\lambda(\omega_0, \tau)| = \sqrt{d^2 + \omega_0^2(\tau)}, \\ \alpha(1+\rho) = \sqrt{S}\tilde{\sigma}(1+\rho) < d, \end{cases}$$

where $\omega_0(\tau)$ is the smallest positive root of the equation $\mathrm{Im}(\lambda(\omega, \tau)) = 0$. Analogous to the analytical results for the predator-prey communities with a discrete time delay, given different values of the parameters, the system exhibits three distinct types of dynamical behaviors: absolutely stable, absolutely unstable, and delay-dependent stability, with respective conditions $(S-1)\mu < d$, $\alpha(1+\rho) > d$, and $\alpha(1+\rho) < d < (S-1)\mu$. Specifically, for the third case, there is a critical time delay $\tau_{\rm cr}$ determined by $(S-1)\mu = |\lambda(\omega_0(\tau_{\rm cr}), \tau_{\rm cr})|$. As the time delay increases from zero and exceeds a certain amount, the maximal capacity of the competitive ecosystems decreases, as shown in Fig. 5**f** of the main text.

In Table S1, we list the results of numerical simulations on the maximal capacities of the mutualistic and competitive ecosystems. The results agree with those calculated directly from the respective theoretical formulas.

5.2 Cascade and niche predator-prey ecosystems

The species in a cascade ecosystem [18] form a unidirectional food chain, i.e., all the species are labeled consecutively by integers $1, \ldots, S$ and species j prey on species with smaller indices, i.e., $1, \ldots, j-1$. There is a top predator with index S and a bottom prey with index 1. With these labeling, all the positive elements $a_{ij} > 0$ are located in the lower triangular part while all negative elements $a_{ij} < 0$ fall in the upper triangular part, see Fig. 5**c** of the main text. The configuration of the cascade predator-prey systems is analogous to that of the random predator-prey ecosystems except that each pair (a_{ij}, a_{ji}) with $i > j$ take values zero with probability $1 - C$ and is drawn independently from $\left(\left|\mathcal{N}(0, \sigma^2)\right|, -\left|\mathcal{N}(0, \sigma^2)\right|\right)$ with probability C.

The ecosystems with niche structure [19, 20] refines the cascade structure by loosening the unidirectional food chain, which is then capable of incorporating more diverse ecological features such as cannibalism, looping and flexible food chain length. In a niche system, each species i is allocated to a niche value n_i uniformly distributed in the interval $[0, 1]$. While $n_i > n_j$ means i preys on j in a cascade system, the difference here is that each species i is further assigned a niche interval $[c_i - r_i/2, c_i + r_i/2]$ with a niche center c_i and range r_i, and only preys on those species j whose niche values n_j are contained within the niche interval of i. The niche range r_i is the product of n_i and a variable following the Beta distribution with the probability density function $\beta(1-x)^{\beta-1}$, where $\beta = 1/C - 1$ is determined by the sparsity parameter C. The niche center c_i is drawn uniformly and independently from the interval $[r_i/2, n_i]$ for each species i.

The configuration results in a binary adjacency matrix $\mathbf{B}$ with elements b_{ij} for the niche structures, where $b_{ji} - b_{ij}$ represents a qualitative relationship from the species j to i. The elements a_{ij} of the community matrix $\mathbf{A}$ is then constructed with its modulus from an absolute Gaussian variable $\left|\mathcal{N}(0, \sigma^2)\right|$ and its sign drawn from $b_{ji} - b_{ij}$.

The distributions the eigenvalues of the cascade and niche matrices do not obey the elliptic law, because the matrix elements are not homogeneously distributed with the identical moment. Numerical computations reveal that, for both types of matrices, the first several eigenvalues are located near the imaginary axis and isolated from the remaining ones that are distributed approximately on an ellipse, as shown in Figs. 5**c**–**d** of the main text. We also find that, for systems with sufficiently large community size, the spectral radii are proportional to S, and the size of the ellipse is proportional to $\sqrt{S}$, a feature of the elliptic law, as shown in Fig. S4.

We analyze the relationship between the stability region and the distribution of eigenvalues. For systems with a cascade or a niche structure without time delay (i.e., $\tau = 0$), the stability depends on the values of d and λ_+, the eigenvalues possessing the largest real part, which is nothing but the length of the semi-horizontal axis of the "ellipse". When time delay is present, for a system with large community size, the stability region is a bounded leaf-shape area and the stability is dominated by the leading eigenvalue $\lambda_{\max}$ (possessing the greatest modulus) rather than λ_+, because the former is of order $\mathcal{O}(S)$ while the latter is of order $\mathcal{O}(\sqrt{S})$. In practice, we are able to compute numerically the maximal capacity $S_{\max}$ when other parameters are fixed (e.g., Table S1 and Fig. 5 of the main text). Because the distributions of the eigenvalues of the two types of interacting structures expand more widely and grow faster than that of the predator-prey communities, the maximal capacities of the former ones are smaller than that of the latter one and decay rapidly as the amount of the time delay τ increases. As a result, a sufficiently large time delay implies that the systems with cascade and niche structures

accommodate even less complexity (or capacity) than the mixed ecosystems, making them the last system in the hierarchical order of complexity. In addition, different distributions of the eigenvalues suggest that the cascade systems accommodate more species than the niche ones. We also simulate the case with distributed time delays with varying time shift $\hat{\tau}$ or average time delay $\langle\tau\rangle$. The results are provided in Figs. 5**h**–**j** of the main text.

5.3 Effect of asymmetric delays in predator-prey type ecosystems

The nature of the predator-prey interaction suggests that the populations of the prey species decrease almost instantaneously even when time delay is present. For asymmetric time delays, the ecosystem is described by

$$\dot{x}_i(t) = -dx_i(t) + \sum_{j:a_{ij}<0} a_{ij}x_j(t) + \sum_{j:a_{ij}>0} a_{ij}x_j(t-\tau), \tag{S36}$$

where $\mathbf{A}$ with elements a_{ij} is the predator-prey type of community matrix, and a negative coefficient $a_{ij} < 0$ means that species j preys on and affects i without time delay. The system can be rewritten in the following matrix form

$$\dot{\boldsymbol{x}}(t) = -d\boldsymbol{x}(t) + \mathbf{A}_{-}\boldsymbol{x}(t) + \mathbf{A}_{+}\boldsymbol{x}(t-\tau),$$

where $\mathbf{A}_{+} = \{\max(a_{ij}, 0)\}$ and $\mathbf{A}_{-} = \{\min(a_{ij}, 0)\}$ are the positive and negative parts of matrix $\mathbf{A}$, respectively.

We focus on three types of systems that contain different predator-prey type of interactions: random predator-prey, cascade and niche structures, and investigate how unidirectional time delays affect the admissible complexity and/or capacity. Due to the heterogeneity of the time delay, a uni-dimensional characteristic equation is not adequate for the analytical investigation. We detect the stability numerically and directly from the simulated trajectories of Eq. (S36). The results are depicted in Fig. S5**a**. Apparently, the asymmetric time delay does not change the hierarchical order of complexity for different predator-prey type ecosystems. Moreover, the asymmetric time delay also mitigates the admissible complexity or capacity of the ecosystems with the same trends as those with bidirectional time delays.

For the three types of ecosystems, we also introduce the concept of effective time delay τ_{eff} that depends on the actual time delay τ. In such a way, the system with bidirectional time delay τ_{eff} shares the same admissible complexity or maximal capacity as the one with unidirectional time delay τ. The values of τ_{eff} can be computed numerically. As shown in Fig. S5**b**, the effective time delay τ_{eff} of the asymmetric systems are much smaller than τ. For the predator-prey ecosystems, the value of τ_{eff} is approximately $\tau/2$, whereas for the systems with cascade and niche structures, the values of τ_{eff} are even less. In spite of the unidirectional time delay structure, the overall tendency is analogous and the hierarchical order of complexity sustains among the different types of predator-prey communities.

6 Estimation of the amount of time delay

We consider a general time-delayed system described by

$$\frac{\mathrm{d}\boldsymbol{x}(t)}{\mathrm{d}t} = \boldsymbol{f}\left(\boldsymbol{x}(t), \boldsymbol{x}_\tau(t)\right), \quad \boldsymbol{x} \in \mathbb{R}^n. \tag{S37}$$

where $\boldsymbol{x}_\tau(t) = \boldsymbol{x}(t-\tau)$. Assume that the system admits a positive equilibrium state $\boldsymbol{x} = \boldsymbol{x}^\star$ satisfying $\boldsymbol{f}(\boldsymbol{x}^\star, \boldsymbol{x}^\star) = \boldsymbol{0}$. To study the dynamics in the vicinity of $\boldsymbol{x}^\star$, we consider the linearization of Eq. (S37) around $\boldsymbol{x}^\star$ and obtain the following equation in terms of $\boldsymbol{y}(t) = \boldsymbol{x}(t) - \boldsymbol{x}^\star$

$$\frac{\mathrm{d}\boldsymbol{y}(t)}{\mathrm{d}t} = \mathcal{D}\boldsymbol{f}_1(\boldsymbol{x}^\star)\boldsymbol{y}(t) + \mathcal{D}\boldsymbol{f}_2(\boldsymbol{x}^\star)\boldsymbol{y}(t-\tau). \tag{S38}$$

where

$$\mathcal{D}\boldsymbol{f}_1(\boldsymbol{x}^\star) = \left.\frac{\partial \boldsymbol{f}}{\partial \boldsymbol{x}}\right|_{\boldsymbol{x}=\boldsymbol{x}^\star, \boldsymbol{x}_\tau=\boldsymbol{x}^\star}, \quad \mathcal{D}\boldsymbol{f}_2(\boldsymbol{x}^\star) = \left.\frac{\partial \boldsymbol{f}}{\partial \boldsymbol{x}_\tau}\right|_{\boldsymbol{x}=\boldsymbol{x}^\star, \boldsymbol{x}_\tau=\boldsymbol{x}^\star}$$

are, respectively, the Jacobian matrices of $\boldsymbol{f}(\boldsymbol{x}, \boldsymbol{x}_\tau)$ about $(\boldsymbol{x}^\star, \boldsymbol{x}^\star)$. According to the theory of functional differential equations [21], the stability of the equilibrium state $\boldsymbol{x}^\star$ is determined by the stability of the zero solution $\boldsymbol{y}(t) \equiv \boldsymbol{0}$ of the system (S38).

Introducing a new time variable s which satisfying the linear transformation $t = Ts$ with $T \in \mathbb{R}^+$ and defining that $\boldsymbol{z}(s) \coloneqq \boldsymbol{y}(Ts)$, we have

$$\boldsymbol{y}(t-\tau) = \boldsymbol{y}(T(s-\tau/T)) = \boldsymbol{z}(s-\tau_0),$$

where $\tau_0 \coloneqq \tau/T$. System (S38) now becomes

$$\frac{\mathrm{d}\boldsymbol{z}(s)}{\mathrm{d}s} = T\cdot\mathcal{D}\boldsymbol{f}_1(\boldsymbol{x}^\star)\boldsymbol{z}(s) + T\cdot\mathcal{D}\boldsymbol{f}_2(\boldsymbol{x}^\star)\boldsymbol{z}(s-\tau_0). \tag{S39}$$

We investigate the relation between the amount of time delay in the linearized equation and the corresponding amount in the original ecological system. We focus on the following S-dimensional generalized Lotka-Volterra system

$$\frac{\mathrm{d}x_i(t)}{\mathrm{d}t} = r_i x_i(t)\left[1 - a_{ii}x_i(t) + \sum_{j=1, j\neq i}^{S} a_{ij}x_j(t-\tau)\right], \quad i = 1, \dots S, \tag{S40}$$

where x_i is the abundance of the ith population, t is time in units of year, month, or day, r_i stands for the natural growth rate of the ith population, a_{ii} is the reciprocal number of the maximal capacity of the ith population, and a_{ij} characterizes the interacting strength from the jth to the ith population. Interactions with $a_{ij} < 0$ are of the predator-to-prey or the competition type, while those with $a_{ij} > 0$ are of the prey-to-predator or mutualistic type. For simplicity, we use the homogeneous time delay τ in system (S40) and assume the existence of a positive equilibrium state denoted by $\boldsymbol{x}^\star = [x_1^\star, \dots, x_N^\star]$. We then obtain a linearized equation of (S40) about the equilibrium state as in Eq. (S38). Note that the linearized system with time delay considered in the main text is written as

$$\frac{\mathrm{d}\boldsymbol{x}(s)}{\mathrm{d}s} = -d\boldsymbol{x}(s) + \mathbf{A}\boldsymbol{x}(s-\tau_0).$$

Unifying the two linearized systems requires that $d\cdot\boldsymbol{I} = -T\cdot\mathcal{D}\boldsymbol{f}_1(\boldsymbol{x}^\star)$, where $\boldsymbol{I}$ is the $S\times S$ identity matrix. For simplicity, we set the damping rate d for every population as the average of the derivatives about the equilibrium state for all populations

$$d \triangleq -T\frac{1}{N}\mathtt{tr}\left[\mathcal{D}\boldsymbol{f}_1(\boldsymbol{x}^\star)\right] = -T\frac{1}{N}\sum_i (r_i - 2r_i a_{ii} x_i^\star),$$

The scaling constant thus becomes

$$T = -\frac{d}{\frac{1}{N}\sum_i (r_i - 2r_i a_{ii}x_i^\star)} = \frac{d}{2\langle r_i a_{ii} x_i^\star\rangle - \langle r_i\rangle},$$

which implies that

$$\tau = T\tau_0 = \frac{d\tau_0}{2\langle r_i a_{ii} x_i^\star \rangle - \langle r_i \rangle},$$

where τ is the original time delay in the system (S40). The values of the vector $\boldsymbol{x}^\star$ depend on the values of the interaction a_{ij}. Because the values of the elements in $\boldsymbol{x}^\star$ and those of the interacting strength can be taken from a bounded interval and we also have $a_{ii}x_i^\star < 1$, it is reasonable to regard the values of τ and $\frac{d\tau_0}{\langle r_i \rangle}$ as having the same order of magnitude. For example, for $\langle r_i \rangle \sim 10\text{year}^{-1}$, we have $\tau \sim 10^{-1}\text{year}$. That is, the amount of time delay is approximately on the order of one month, which is consistent with the practical data in the survey [22] indicating that the estimated amount of time delay for insects is on the order of one month.

7 Supplementary table

	$S_{\max}$ (Discrete time delay)			$S_{\max}$ (Gamma-distributed time delay)		
	$\tau = 0$	$\tau = 0.165$	$\tau = 0.5$	$\hat{\tau} = 0$	$\hat{\tau} = 0.083$	$\hat{\tau} = 0.5$
Mutualism	35	35	35	35	35	35
Competition	83	82	63	83	83	59
Mixture	69	69	69	69	69	69
Random	184	181	178	182	181	177
Predator-prey	1333	185	100	305	184	95
Cascade	1180	87	60	118	87	58
Niche	522	61	44	81	61	42

Table S1: Empirical maximal capacities for different types of ecosystems with or without time delays. The maximal capacity $S_{\max}$ are obtained by numerical simulations and the probit model. The values of the discrete time delays are 0, 0.165, 0.5. For the distributed time delay, we consider the Gamma distribution with $\theta = 0.05$ and $m = 2$ with time shift $\hat{\tau} = 0$, 0.083 and 0.5. Other parameters used here are $d = 3$, $\sigma = 0.5$ and $C = 0.2$.

8 Supplementary figures

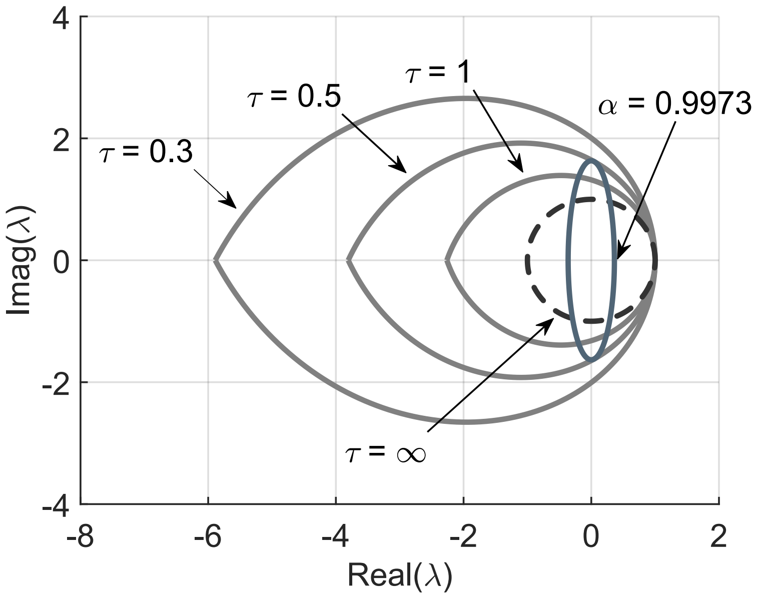

Supplementary Fig. S1: **Boundary of the stability region and distribution of eigenvalues.** The gray curves are the boundaries of the stability region when there is a discrete time delay $\tau = 0.3,\ 0.5$ and 1. The dashed black circle is the limiting case when $\tau \to \infty$. The circle is centered at the origin with radius d. The blue curve indicates the distribution of eigenvalues of the community matrix $\mathbf{A}$ for the predator-prey communities corresponding to a negative correlation ρ. It is the system with critical admissible complexity $\alpha^* = 0.9973$ when there is a time delay $\tau = 0.5$. We see that the ellipse intersects with the stability region. Other parameters used here are $d = 1$, $\rho = -2/\pi$.

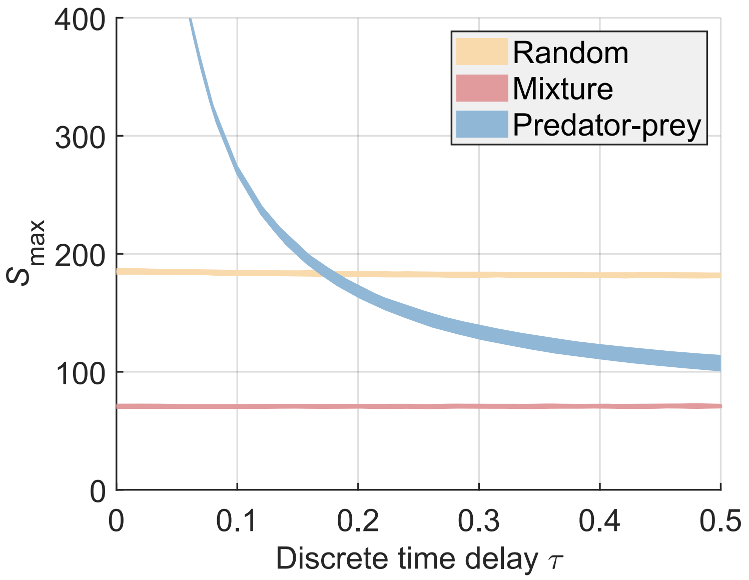

Supplementary Fig. S2: **Robustness of the complexity hierarchy**. The maximal capacity $S_{\max}$ of the three representative ecosystems with heterogeneous time delay are shown. The time delays τ_{ij} are sampled from a uniform distribution with mean ranging from 0.02 to 0.5 and coefficient of variation (cv) ranging from 0.05 to 0.5. The regions bounded by the mean ± standard deviation of the maximal capacity are filled among different values of cv with respect to τ. Other parameters used here are $d = 3$, $C = 0.2$, and $\sigma = 0.5$.

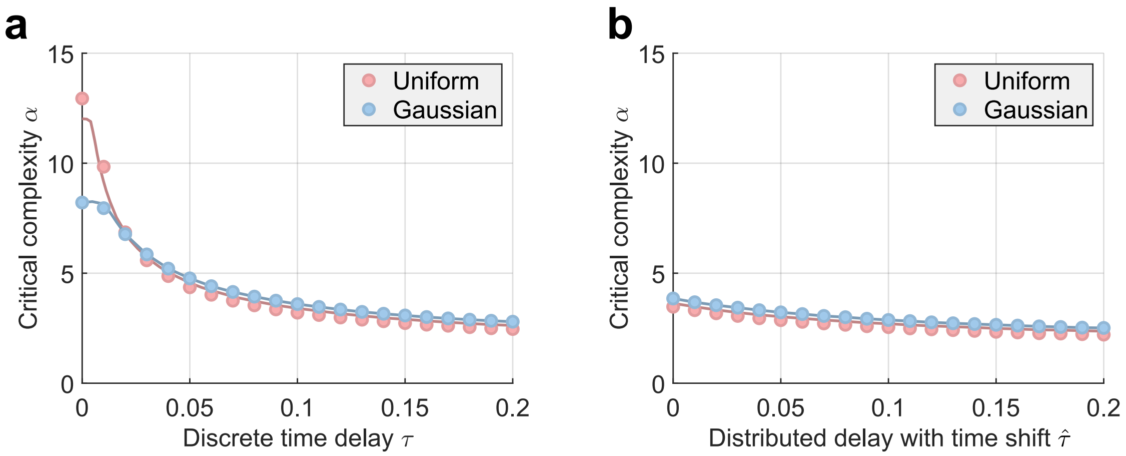

Supplementary Fig. S3: **Comparisons of critical complexity α^* for different ensemble distributions in predator-prey ecosystems**. **a**, For each given value of a discrete time delay τ, the critical complexity α^* is estimated from both theoretical analysis (solid curves) and numerical simulations (dots). The elements a_{ij} are drawn from a Gaussian (blue) or a uniform (red) distribution. **b**, The results when considering the distributed time delay (Gamma distribution) with time shift $\hat{\tau}$. Parameters used here are $S = 400$, $C = 0.1$, and $d = 3$.

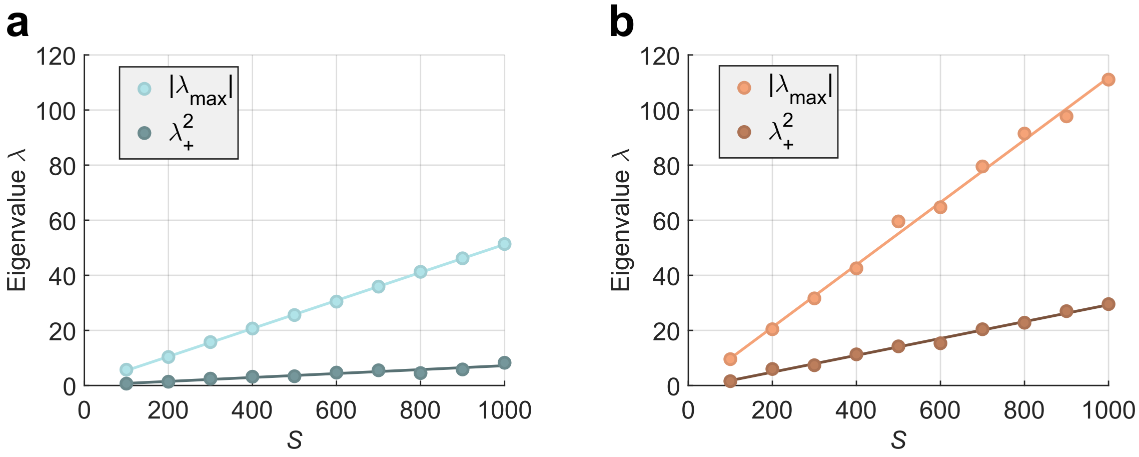

Supplementary Fig. S4: **Properties of eigenvalues versus the community size for cascade and niche structures**. **a** and **b** show the largest eigenvalues of the community matrix **A** of the predator-prey ecosystems with cascade and niche structure, respectively. Light and dark dots are respectively the eigenvalues with largest modulus ($|\lambda_{\max}|$) and the square of the largest real part (λ_+^2). Solid lines are linear fittings with $|\lambda_{\max}| \sim \mathcal{O}(S)$ and $\lambda_+ \sim \mathcal{O}(\sqrt{S})$ for both cases. Parameters used here are $C = 0.2$, $\sigma = 0.5$, and $S = 100,\ 200, \cdots, 1000$.

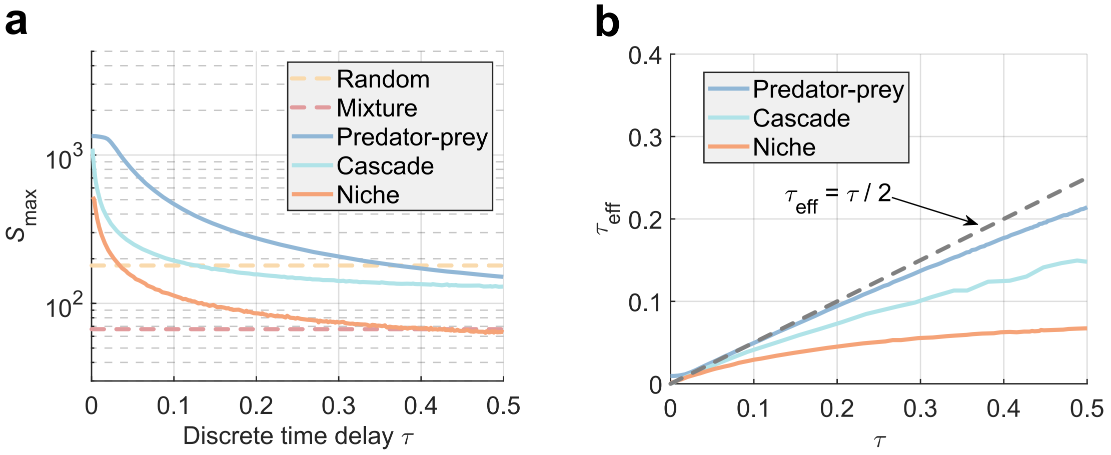

Supplementary Fig. S5: **Predator-prey type ecosystems with asymmetric time delays.** **a**, Numerically computed maximal capacity $S_{\max}$ for the three predator-prey type ecosystems with asymmetric time delay. Systems with (random) predator-prey, cascade and niche structures are shown in blue, cyan and orange, respectively. The results of random (yellow dashed) and mixed ecosystems (red dashed) are also presented for comparison. The monotonic decreasing of $S_{\max}$ for the three ecosystems are analogous to those for the counterparts with bidirectional time delays. Moreover, the hierarchical order of the three predator-prey type ecosystems sustains. **b**, For each value of asymmetric time delay τ, the corresponding effective time delay τ_{eff} of the systems with bidirectional time delays is calculated from $S_{\max}$. All three curves are located below the line $\tau_{\text{eff}} = \tau/2$. The parameters used here are $d = 3$, $C = 0.2$, and $\sigma = 0.5$.

References

[1] J. Feinberg and A. Zee. Non-hermitian random matrix theory: Method of hermitian reduction. *Nuc. Phys. B*, **504**:579–608, 1997.

[2] M. Timme, F. Wolf, and T. Geisel. Topological speed limits to network synchronization. *Phys. Rev. Lett.*, **92**:074101, 2004.

[3] M. Timme, T. Geisel, and F. Wolf. Speed of synchronization in complex networks of neural oscillators: analytic results based on random matrix theory. *Chaos*, **16**, 2006.

[4] J. W. Baron, T. J. Jewell, C. Ryder, and T. Galla. Eigenvalues of random matrices with generalized correlations: A path integral approach. *Phys. Rev. Lett.*, **128**:120601, 2022.

[5] V. L. Girko. Circular law. *Theor. Prob. Appl.*, **29**:694–706, 1985.

[6] F. Götze and A. Tikhomirov. The circular law for random matrices. *Ann. Prob.*, pages 1444–1491, 2010.

[7] T. Tao, V. Vu, and M. Krishnapur. Random matrices: Universality of esds and the circular law. *Ann. Prob.*, pages 2023–2065, 2010.

[8] V. L. Girko. Elliptic law. *Theor. Prob. Appl.*, **30**:677–690, 1986.

[9] H. J. Sommers, A. Crisanti, H. Sompolinsky, and Y. Stein. Spectrum of large random asymmetric matrices. *Phys. Rev. Lett.*, **60**:1895, 1988.

[10] A. Naumov. Elliptic law for real random matrices. *arXiv preprint arXiv:1201.1639*, 2012.

[11] H. H. Nguyen and S. O'Rourke. The elliptic law. *Int. Math. Res. Notices*, **2015**:7620–7689, 2015.

[12] F. Götze, A. Naumov, and A. Tikhomirov. On minimal singular values of random matrices with correlated entries. *Random Matrices: Theory Appl.*, **4**:1550006, 2015.

[13] M. L. Mehta. *Random matrices.* Elsevier, 2004.

[14] V. K. Jirsa and M. Ding. Will a large complex system with time delays be stable? *Phys. Rev. Lett.*, **93**:070602, 2004.

[15] Y. Yang, K. R. Foster, K. Z. Coyte, and A. Li. Time delays modulate the stability of complex ecosystems. *Nat. Ecol. Evol.*, **7**:1610–1619, 2023.

[16] S. A. Campbell and R. Jessop. Approximating the stability region for a differential equation with a distributed delay. *Math. Mod. Nat. Phen.*, **4**:1–27, 2009.

[17] S. Allesina and S. Tang. Stability criteria for complex ecosystems. *Nature*, **483**:205–208, 2012.

[18] A. R. Solow and A. R. Beet. On lumping species in food webs. *Ecology*, **79**:2013–2018, 1998.

[19] R. J. Williams and N. D. Martinez. Simple rules yield complex food webs. *Nature*, **404**:180–183, 2000.

[20] T. Gross, L. Rudolf, S. A. Levin, and U. Dieckmann. Generalized models reveal stabilizing factors in food webs. *Science*, **325**:747–750, 2009.

[21] J. K. Hale. Theory of functional differential equations, volume 3 of. *Applied Mathematical Sciences*, 4, 1977.

[22] N. MacDonald. Biological delay systems: Linear stability theory. 1989. *Cambridge Studies in Mathematical Biology*, 1989.
